# Efficacy and safety of tumor necrosis factor-α inhibitors and mesenchymal stem cells in the treatment of fistulizing Crohn’s disease: a systematic review and network meta-analysis

**DOI:** 10.3389/fimmu.2026.1929235

**Published:** 2026-09-11

**Authors:** Xue-Min Chen, Li-Chun Han, Bing Han, Jianing Lin, Liyan Chen, Liumei Yan, Yufeng Huang, Shiquan Li, Xiaoping Lv

**Affiliations:** 1 Department of Gastroenterology, The First Affiliated Hospital, Guangxi Medical University, Nanning, China; 2 Department of Gastroenterology, The First Affiliated Hospital of Zhengzhou University, Zhengzhou, China; 3 Department of Gastroenterology, Wuzhou Red Cross Hospital, Wuzhou, Guangxi, China

**Keywords:** Crohn’s disease, mesenchymal stem cells, network meta-analysis, perianal fistula, tumor necrosis factor-α inhibitors

## Abstract

**Background:**

Tumor necrosis factor-α inhibitors (anti-TNF-α) and mesenchymal stem cells (MSCs) have been proven to be effective for Perianal fistulizing Crohn’s disease (PFCD). However, no head-to-head clinical trials have directly comparing these two therapies. Therefore, using a network meta-analysis and systematic review, we evaluated the efficacy and safety of anti-TNF-α and MSCs in PFCD patients.

**Methods:**

We included randomized controlled trials (RCTs) evaluating anti-TNF-α or MSCs against placebo (PBO) in PFCD patients. The primary efficacy outcomes were fistula remission and fistula response. The secondary outcome was clinical remission. Safety outcomes comprised adverse events and infections. Effect sizes were reported as odds ratios (ORs) with 95% credible intervals (CrIs). Treatment rankings were determined using the surface under the cumulative ranking curve (SUCRA).

**Results:**

A total of 15 RCTs involving 2, 475 patients were included. Compared with PBO, MSCs significantly improved fistula remission (OR = 5.33, 95% CrI: 2.19–19.03) and fistula response (OR = 4.76, 95% CrI: 1.78–20.37). Anti-TNF-α also significantly increased fistula remission rates (OR = 2.19, 95% CrI: 1.01–4.54). SUCRA rankings placed MSCs first for both fistula remission (SUCRA = 0.97) and fistula response (SUCRA = 0.967). For clinical remission, anti-TNF-α demonstrated superior efficacy (OR = 2.41, 95% CrI: 1.30–5.03) compared with MSCs (OR = 1.71, 95% CrI: 0.69–5.57) and PBO. Indirect comparisons revealed a non-significant trend favoring MSCs over anti-TNF-α for fistula remission (OR = 2.44) and fistula response (OR = 2.55). Neither treatment showed statistically significant differences in adverse events or infections versus PBO, indicating favorable safety profiles for both.

**Conclusion:**

In indirect comparisons, MSCs showed a numerically greater but not statistically significant effect on fistula healing compared with anti-TNF-α, whereas anti-TNF-α was superior for clinical remission. Both treatments were generally safe. Given the indirect nature of these comparisons, direct head-to-head trials are needed to establish their comparative efficacy.

**Systematic Review Registration:**

https://www.crd.york.ac.uk/PROSPERO/recorddashboard, identifier CRD42021283052.

## 1 Introduction

Perianal fistulizing Crohn’s disease (PFCD) represents one of the most prevalent and clinically burdensome complications of Crohn’s disease (CD). The cumulative incidence of PFCD is 8.3%-11% within the first year following CD diagnosis. These manifestations result in significant functional impairment and greatly impact the patients’ quality of life (1). The management of PFCD primarily relies on a combined strategy of pharmacological therapies and surgical intervention (2, 3). Regardless of a range of applied strategies, the overall recurrence after initial healing was known to remain high at 37-44% (4, 5).

Long-term medical therapy remains a cornerstone of treatment. The advent of biologics has significantly improved clinical symptoms and achieved fistula healing, with tumor necrosis factor-α inhibitors (anti-TNF-α) established as the cornerstone of medical treatment for PFCD over the past two decades (6, 7). To date, several additional RCTs confirmed the clinical efficacy of anti-TNF-α for induction and maintenance therapy of PFCD (8–15), Vuyyuru et al. (16) demonstrated that when limited to studies with fistula response as the primary endpoint, anti-TNF-α was significantly superior to placebo (RR = 1.94, 95% CI: 1.10–3.41). However, anti-TNF-α has limitations: approximately 50% to 60% of patients failed to achieve complete fistula closure after the induction phase, long-term remission rates remain low, and relapse rates are high upon drug discontinuation (8, 17). Even with anti-TNF-α treatment, a substantial proportion of PFCD patients still face inadequate therapeutic responses or difficulties in maintaining long-term remission (18).

Recently, the application of stem cells in biomedicine is getting more extensive, these innovative therapies are mainly based on mesenchymal stem cell (MSC) which possesses trophic, angiogenic, anti-inflammatory, and anti-fibrotic activity (19–21). There are multiple studies that have assessed the efficacy and safety of Mesenchymal Stem Cells (MSCs) for PFCD patients (22–28), some clinical trials have indicated that MSCs achieve higher remission rates than PBO in the treatment of PFCD (24, 29). However, the place of MSCs in practice is not yet clear, as few randomized controlled trials(RCTs) are available, and the comparative effectiveness of the different strategies is difficult to establish.

Although both anti-TNF-α and MSCs have been proven to be effective treatments for PFCD, there is currently a lack of head-to-head clinical trials directly comparing these two therapies. Consequently, there remains considerable uncertainty regarding how to select or sequentially administer them in clinical practice. Traditional pairwise meta-analyses can only compare individual interventions against PBO; they cannot simultaneously rank or directly compare different categories of interventions (such as anti-TNF versus MSCs from various sources or at different dosages) within a unified framework. Network meta-analysis (NMA), an extension of traditional meta-analysis, integrates both direct and indirect evidence to simultaneously compare multiple interventions and estimate the probability of each being the optimal treatment.

To our knowledge, this is the first Bayesian network meta-analysis to systematically compare the efficacy of anti-TNF-αand MSC therapies for PFCD. Our findings are expected to not only delineate the optimal treatment strategy but also provide actionable evidence for personalized management and highlight critical directions for future comparative effectiveness research.

## 2 Methods

This study was conducted in accordance with Cochrane criteria, the Preferred Reporting Items for Systematic Reviews and Meta-Analyses (PRISMA) statement (30) and relevant meta-analysis guidance. The study protocol was registered with PROSPERO (CRD42021283052), and no amendments to the protocol were made after registration.

### 2.1 Data sources and search strategy

The following electronic databases were systematically searched to identify randomized controlled trials (RCTs) evaluating anti-TNF-α and MSCs for fistulizing Crohn’s disease: Web of Science, Scopus, Embase, Clinical Trials.gov, PubMed, Cochrane Library, and CNKI. The search spanned from the inception of each database or registry to December 2025. A combination of Medical Subject Headings and free-text terms was employed. The primary search terms included: “Crohn disease, “ “perianal fistula, “ “tumor necrosis factor inhibitors, “ “mesenchymal stem cells, “ and “randomized controlled trial.” Detailed search strategies are provided in **Supplementary File 1**. Additionally, the reference lists of included studies and relevant reviews were manually screened to supplement any potentially missed literature.

### 2.2 Selection criteria

According to the following criteria, studies were chosen for inclusion (PICOS):① Participants: We restricted adults (>16 years) who meet the standard clinical, radiographic, and endoscopic criteria for active CD and have draining fistulas at baseline (31), to ensure diagnostic homogeneity and a measurable baseline disease activity; ② Intervention: The intervention group received treatment with either anti-TNF-α or MSCs from various sources. Specifically, the anti-TNF-α included infliximab, adalimumab, certolizumab pegol, and golimumab, with no restrictions on dosage or administration frequency. For MSCs, there were no restrictions on the source, cell type, or route of administration. ③ Comparison: The control group received PBO or active comparator drugs, so that we maintain a star-shaped network structure, enabling robust indirect comparisons via a common comparator, which compensates for the absence of head-to-head trials. ④ Outcome: The primary efficacy outcomes were induction of fistula remission and induction of fistula response. Fistula remission was defined as the complete cessation of drainage upon clinical evaluation following short-term intensive intervention. Fistula response was defined as a reduction in fistula drainage and alleviation of inflammation following short-term intervention, commonly defined as a ≥50% reduction in drainage (16, 32). The secondary outcome was a CDAI score of <150, which reflects overall disease activity (33). Safety outcomes included the incidence of treatment-related adverse events (TRAEs) and the occurrence of serious infections (33). ⑤ Study design:Only randomized controlled trials (RCTs) were included, regardless of whether blinding was employed, to minimize selection bias and confounding by indication.

The following studies were excluded: ①Non-RCTs; ②studies lacking extractable outcomes; ③duplicate publications; ④studies with incomplete data; ⑤animal experiments, or *in vitro* studies.

### 2.3 Data extraction and quality assessment

All data were extracted and confirmed by two authors. Extracted data included first author, year of publication, country, disease distribution, intervention parameters (drug, dosage, and schedule), definitions of induction and maintenance of outcomes, total number of PFCD patients in intervention and control groups, proportion of patients who achieved the outcome of interest, and adverse events. No data conversion was required as all outcomes were dichotomous and reported as event counts per group. No imputation methods were used for missing data; all analyses were conducted based on available case data. For studies with multiple follow-up time points, we extracted data at the time point that best corresponded to the definition of induction of response/remission, as prespecified in the protocol. To avoid a carryover effect in randomized crossover studies, we solely retrieved data from the first part of the investigation. Any data extraction discrepancies were handled by discussion with a third reviewer. The Cochrane Collaboration’s recommended risk of bias tool (ROB 2.0), a domain-based assessment (+ indicating low risk of bias; − indicating high risk of bias; and? indicating unclear risk of bias), was utilized by two reviewers to independently evaluate the methodological quality of eligible RCTs. No automation tools were used in the risk of bias assessment process.

### 2.4 Data synthesis

NMA was conducted using a random-effects model within a Bayesian framework, based on the Gemtc package in R (version 4.4.1) (34). Dichotomous outcomes were expressed as odds ratios (ORs) with 95% credible intervals (CrIs). Due to the star-shaped network structure with placebo as the sole common comparator, closed loops were absent, precluding node-splitting for consistency testing. Indirect comparisons were therefore conducted under the assumption of global consistency. Interventions were ranked using the surface under the cumulative ranking curve (SUCRA), where higher values indicate better rankings (35). Publication bias was assessed via comparison-adjusted funnel plots, with an Egger’s test P-value > 0.05 indicating no significant bias (36).

### 2.5 Statistical analysis

Parameter estimation was performed using the Markov chain Monte Carlo (MCMC) method via JAGS (version 4.3.2). The simulation was configured with 4 chains, a burn-in of 20, 000 iterations, followed by 50, 000 further iterations with a thinning interval of 10. Convergence was assessed using the potential scale reduction factor (PSRF), with PSRF < 1.05 indicating convergence (37). The I^2^ statistic was used to measure statistical heterogeneity, with values > 50% indicating significant heterogeneity. Fixed or random-effects meta-analysis was performed depending on heterogeneity (I^2^ < 50%: fixed-effects; I^2^ ≥ 50%: random effect) (38, 39).

## 3 Results

### 3.1 Search results

**Figure 1:**
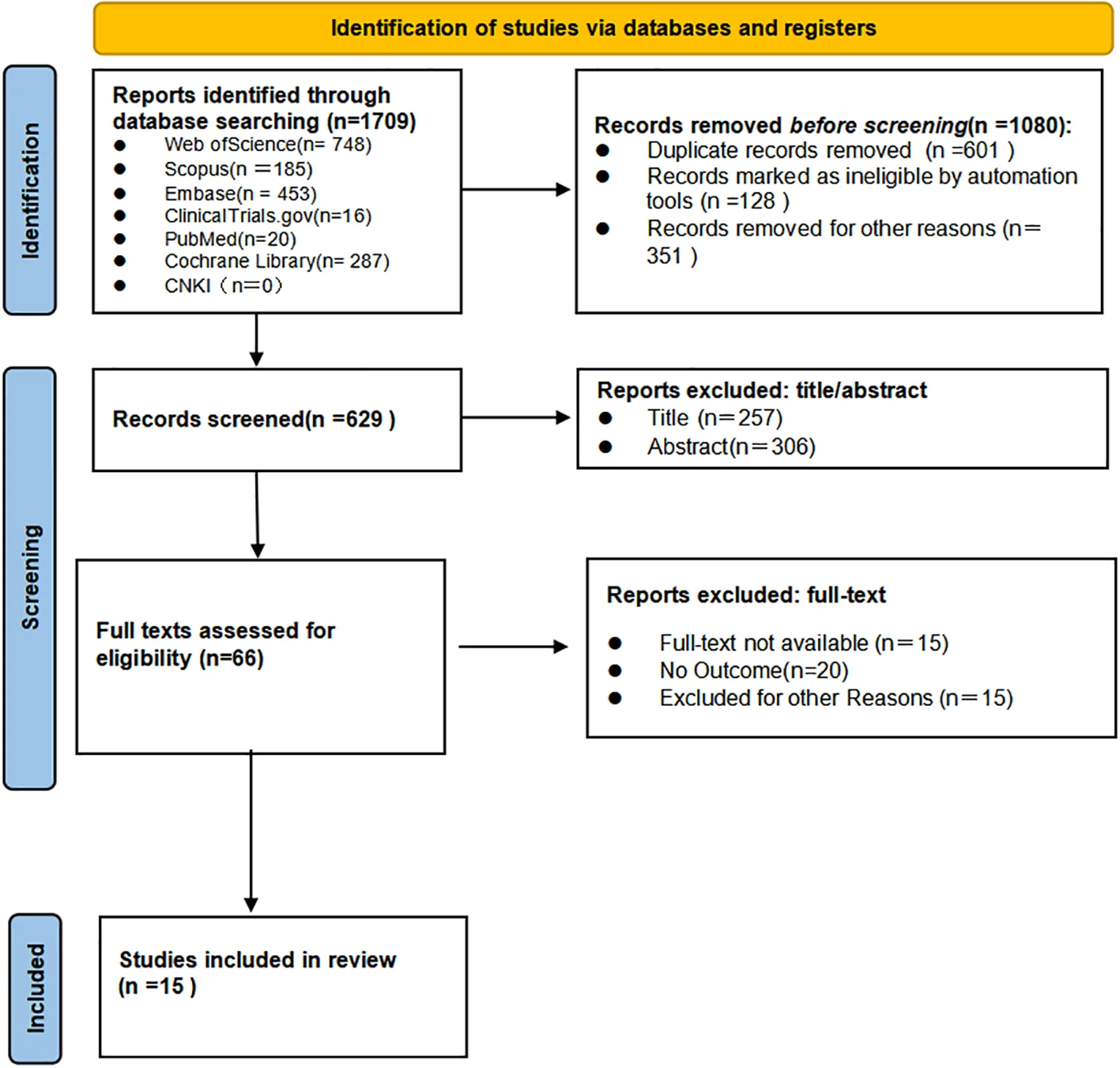
Summary of the evidence search and selection procedure (flow diagram).

Among the 1709 articles yielded from the systematic search, 629 were found to be unique. After reviewing their titles and abstracts, only 66 articles underwent full-text review. Finally, 15 RCTs met the NMA inclusion criteria (**Figure 1**).

### 3.2 Characteristics of included studies

A total of 15 randomized controlled trials involving 2, 475 patients with fistulizing Crohn’s disease were included. Among them, 7 trials (n=412) evaluated MSCs, and 8 trials (n=2, 063) evaluated biologics. Detailed information on study characteristics (**Table 1**), MSC intervention details (**Table 2**), anti-TNF-α details (**Table 3**), and baseline characteristics (**Supplementary Table 1**) is provided in the respective tables. Overall, baseline characteristics were well-balanced across groups. Differences in interventions primarily involved the cell source and dosage of MSCs, as well as the type and administration route of biologics.

**Table 1:** Characteristics of included randomized controlled trials.

Study	Countries; no.of centers	Intervention group	Control group	Fistula type	Follow-up endpoint	Trial design
Garcia-Olmo (2009) (22)	Spain; 1 site	MSCs	PBO	Perianal fistula, Rectovaginal fistula	52 weeks	Indirect ^a^
Molendijk(2015) (23)	Netherlands; 1 site	MSCs	PBO	Perianal fistula	12 weeks	direct
Panés(2016) (24)	Multicenter; 49 sites	MSCs	PBO	Perianal fistula	24 weeks	direct
Lightner(2023I) (25)	USA; 1 site	MSCs	PBO	Perianal fistula, Rectovaginal fistula	24weeks	direct
Lightner(2023II) (26)	USA; 1 site	MSCs	PBO	Perianal fistula	24 weeks	direct
Lightner(2024) (27)	USA;1 site	MSCs	PBO	Rectovaginal fistula	24 weeks	direct
Barnhoorn(2019) (28)	Netherlands, 1 site	MSCs	PBO	Perianal fistula	4 years	NA ^b^
Present(1999) (15)	Multicenter; 12 sites	IFX	PBO	Perianal fistula, Abdominal fistula	18 weeks	direct
Sandborn(2004) (9)	Multicenter; 68 sites	CDP571	PBO	Perianal fistula	28 weeks	subgroup ^c^
Sands(2004) (8)	Multicenter; 45 sites	IFX	PBO	Perianal fistula, Rectovaginal fistula, Abdominal fistula	54 weeks	subgroup
Hanauer(2006) (12)	Multicenter; 55 sites	ADA	PBO	Enterocutaneous fistula, Perianal fistula	4 weeks	subgroup
Sandborn(2007I) (10)	Multicenter; 52 sites	ADA	PBO	Perianal fistula, Abdominal fistula	4 weeks	subgroup
Sandborn(2007II) (11)	Multicenter; 171 sites	CZP	PBO	Perianal fistula, Abdominal fistula	26 weeks	subgroup
Colombel(2009) (13)	Multicenter; 92 sites	ADA	PBO	Perianal fistula, Abdominal fistula	56 weeks	direct
Schreiber(2011) (14)	Multinational; number of centers not specified	CZP	PBO	Perianal fistula	26 weeks	direct

^a^ indicates that the study was not primarily designed for perianal fistula outcomes but reported subgroup data for PFCD patients.

^b^ indicates that the information was not available from the published report.

^c^ indicates that the reported data were derived from a pre-specified subgroup analysis of the original trial.

ADA, adalimumab; CZP, certolizumab pegol; IFX, infliximab; MSC, mesenchymal stem cell; PBO, placebo; PFCD, perianal fistulizing Crohn’s disease.

**Table 2:** Details of RCTs involving MSCs.

Study	Type of MSCs	Source of MSCs	Dose of locally injected MSCs
Garcia-Olmo(2009) (22)	autologous	adipose-derived	First injection of 2.0×10^7^ cells; if no remission at week 8, a second injection of 4.0×10^7^ cells
Molendijk(2015) (23)	allogeneic	bone marrow-derived	Group 1: 1.0×10^7^ cells Group 2: 3.0×10^7^ cells Group 3: 9.0×10^7^ cells
Panés(2016) (24)	allogeneic	adipose-derived	1.2×10^8^ cells
Lightner(2023I) (25)	allogeneic	bone marrow-derived	First injection of 7.5×10^7^ cells; if no remission at 3 months, a second injection of 7.5×10^7^ cells
Lightner(2023II) (26)		bone marrow-derived	First injection of 7.5×10^7^ cells; if no remission at 3 months, a second injection of 7.5×10^7^ cells
Lightner(2024) (27)	allogeneic	bone marrow-derived	7.5×10^7^ cells
Barnhoorn (2019) (28)	allogeneic	bone marrow-derived	Group 1: 1.0×10^7^ cells Group 2: 3.0×10^7^ cells Group 3: 9.0×10^7^ cells

**Table 3:** Details of TNF-α inhibitor-related RCTs.

Study ID	Type of biologic agent	Route of administration	Therapeutic dose
Present(1999) (15)	IFX	IV infusion	induction phase: weeks 0, 2, and 6 (5 mg/kg or 10 mg/kg)
Sandborn(2004) (9)	CDP571	IV infusion	induction phase: week 0 (10 mg/kg) maintenance phase: every 8 weeks (10 mg/kg)
Sands(2004) (8)	IFX	IV infusion	induction phase: weeks 0, 2, and 6 (5 mg/kg) maintenance phase: weeks 14, 22, 30, 38, and 46 (5 mg/kg)
Hanauer(2006) (12)	ADA	SC injection	induction phase: injections at weeks 0 and 2 (40 mg, 80 mg, or 160 mg, with the dose at week 2 reduced by half)
Sandborn(2007I) (10)	ADA	SC injection	induction phase: weeks 0 and 2 (160 mg, with the dose at week 2 reduced by half)
Sandborn(2007II) (11)	CZP	SC injection	induction phase: weeks 0, 2, and 4 (400 mg) maintenance phase: every 4 weeks (400 mg)
Colombel(2009) (13)	ADA	SC injection	induction phase: week 0 (40 mg or 80 mg) maintenance phase: every 1 week or every 2 weeks (40 mg)
Schreiber(2011) (14)	CZP	SC injection	induction phase: weeks 0, 2, and 4 (400 mg) maintenance phase: every 4 weeks (400 mg)

### 3.3 Risk of bias and heterogeneity

**Figure 2:**
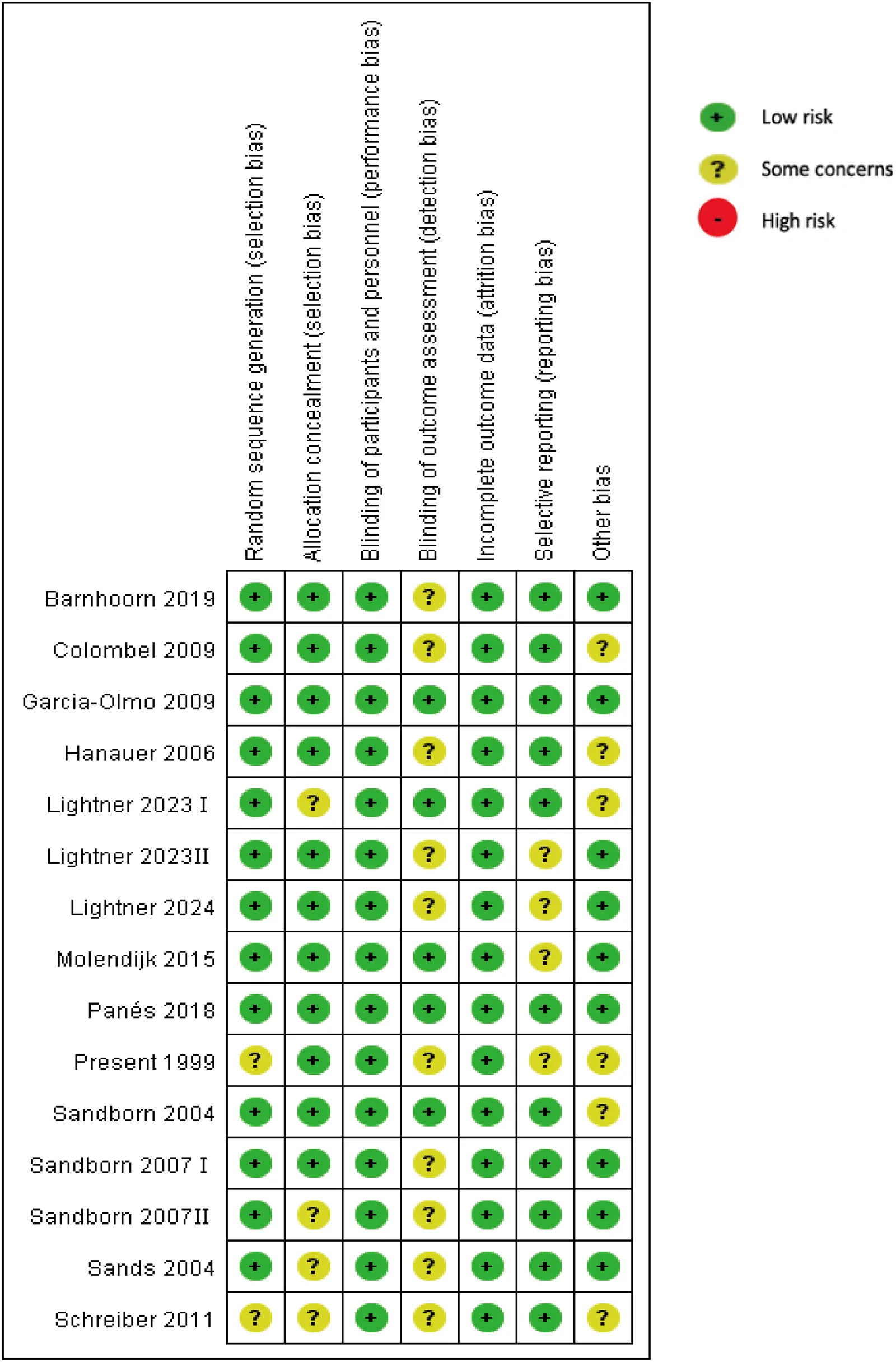
Summary of risk of bias.

**Figure 3:**
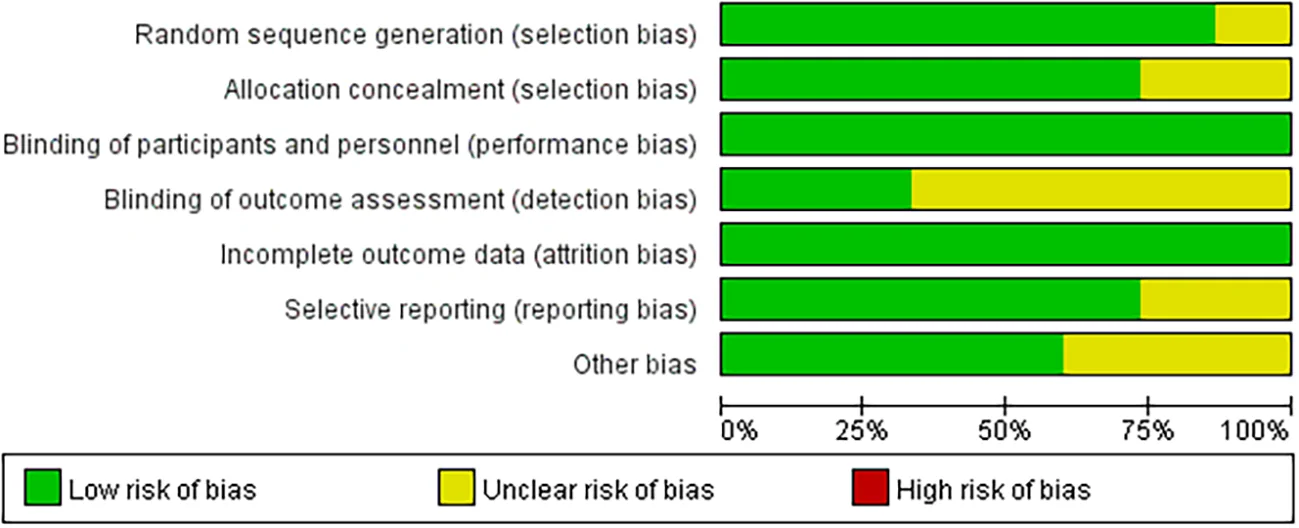
Risk of bias graph.

**Figure 4:**
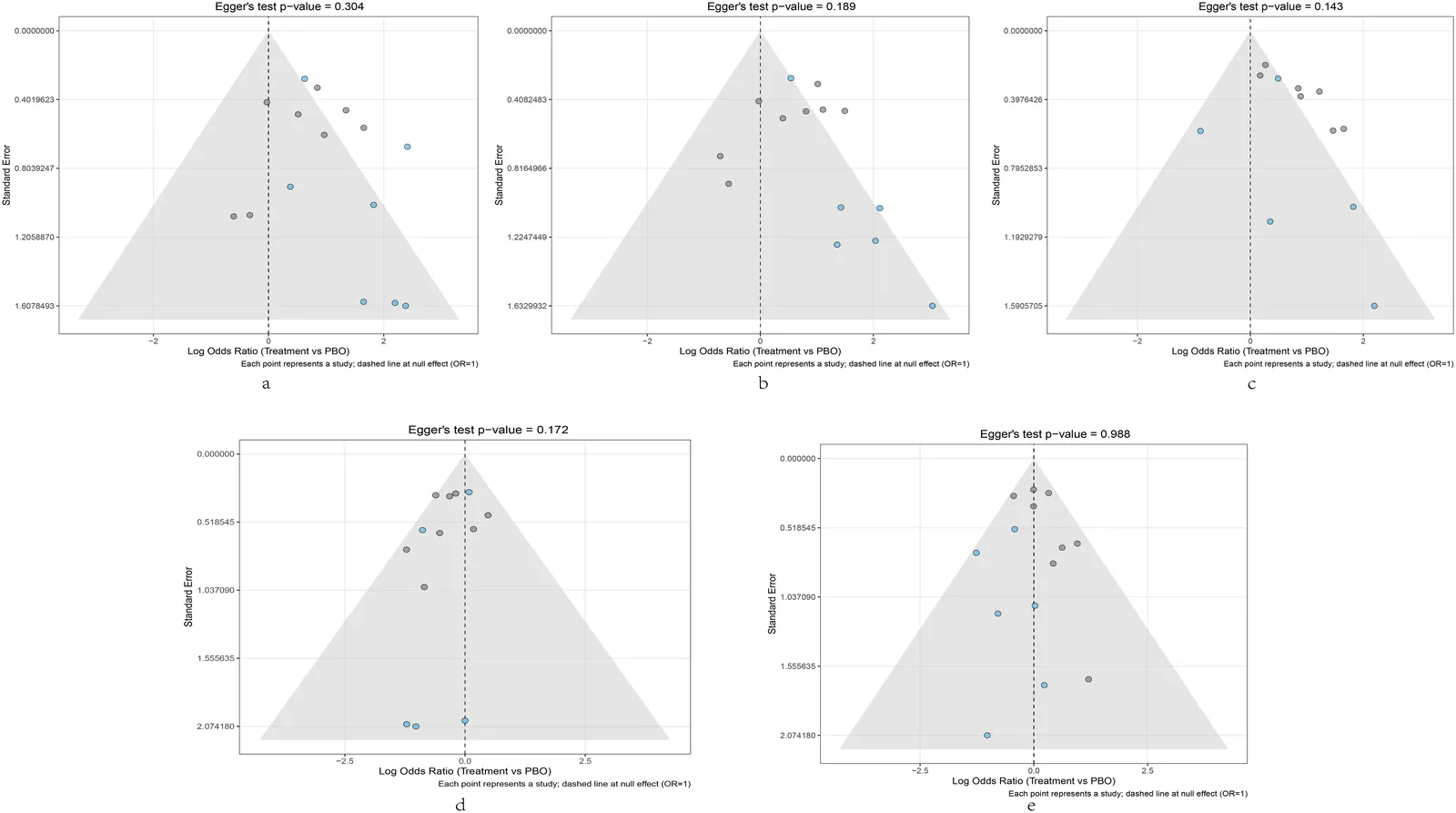
Funnel plots [**(a)** fistula remission; **(b)** fistula response; **(c)** clinical remission; **(d)** adverse events; **(e)** infection rate].

Assessed by the Cochrane Risk of Bias tool, the risks for random sequence generation and allocation concealment were moderate. The overall quality of the 15 included studies was moderate (**Figures 2**, **3**). Funnel plots indicated no significant publication bias (**Figure 4**). Heterogeneity analysis showed low heterogeneity in fistula remission for anti-TNF-α vs PBO (I² = 32.5%) and moderate heterogeneity for MSCs vs PBO (I² = 72.8%). The overall between-study standard deviation (τ) of the network was 0.66 (95% CrI: 0.23–1.52), indicating moderate between-study variability. Given this moderate heterogeneity, a random-effects model was uniformly applied for the network meta-analysis to provide more conservative effect estimates (**Supplementary Table 2**: Heterogeneity Results of the Meta-analysis).

### 3.4 Network geometry

**Figure 5:**
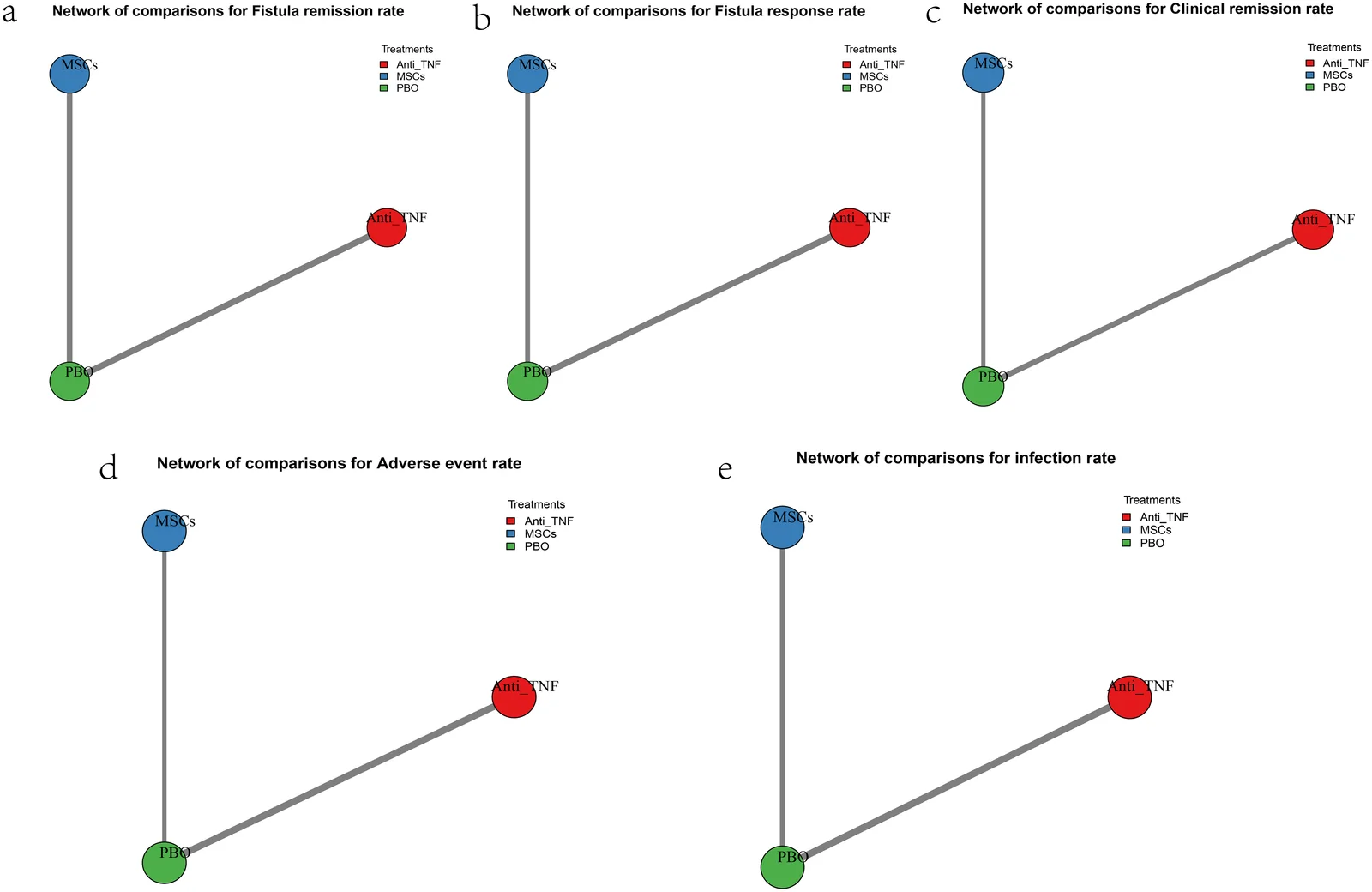
Network geometry [**(a)** fistula remission; **(b)** fistula response; **(c)** clinical remission; **(d)** adverse events; **(e)** infection rate].

**Figure 5** illustrates the network geometry for five outcomes. The network includes two major treatment categories and three interventions (anti-TNF-α, MSCs, PBO), comprising 15 RCTs with 2, 475 patients. Direct comparisons are centered on PBO. Line thickness between nodes indicates the number of included studies, which is comparable between both active interventions and PBO.

### 3.5 Primary outcomes: fistula remission and response induction

**Figure 6:**
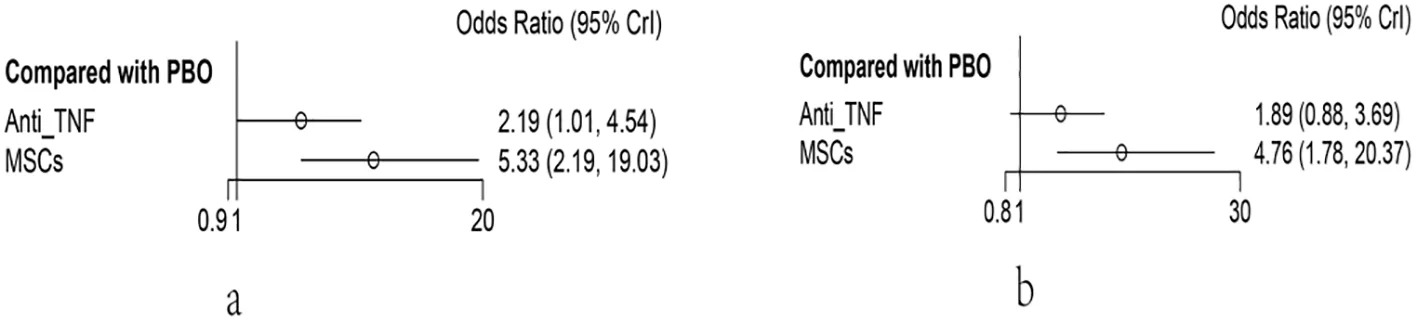
Forest plot [**(a)** fistula remission; **(b)** fistula response].

**Figure 7:**
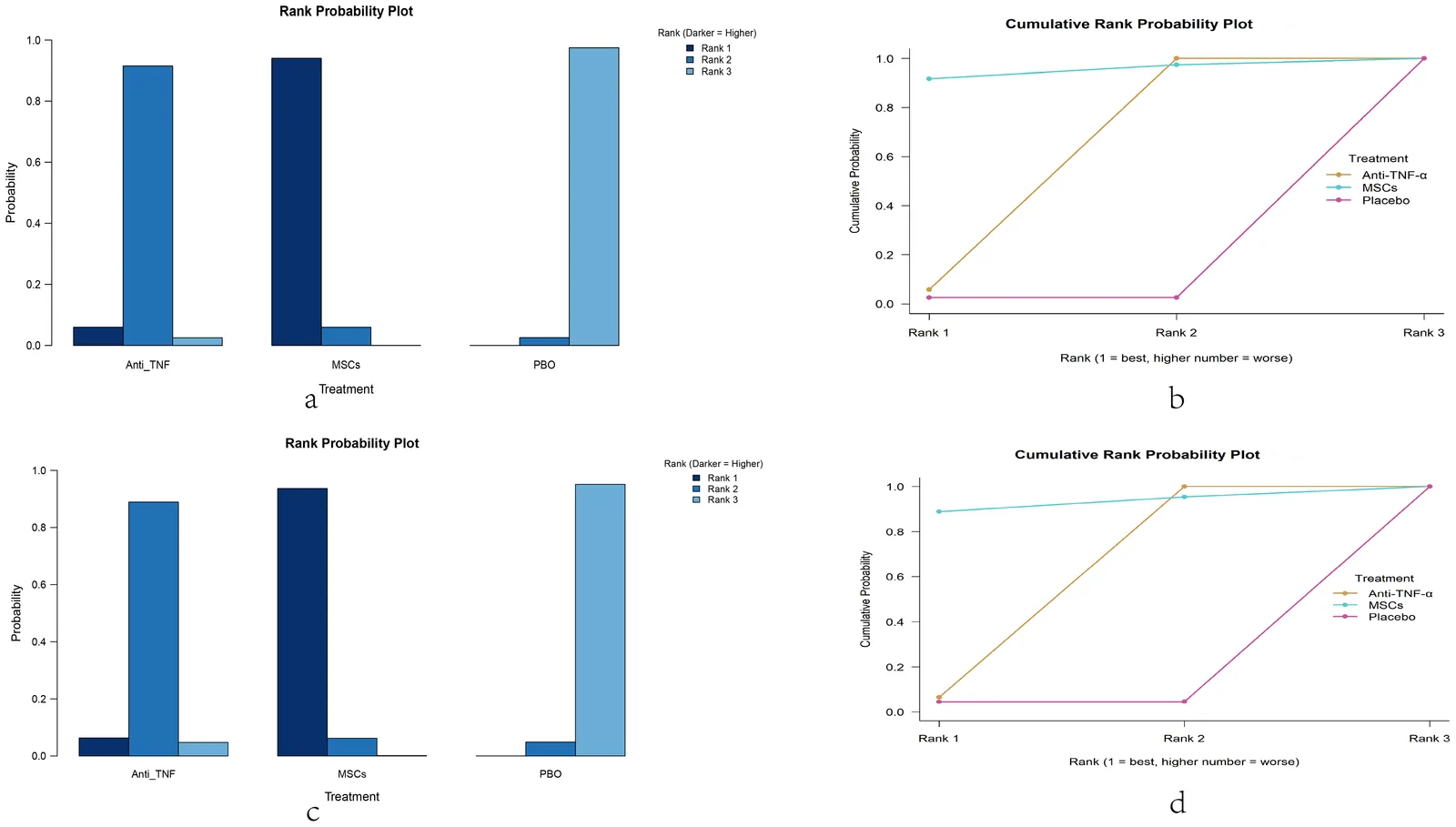
SUCRA ranking plots [**(a)** Bar chart for fistula remission; **(b)** Line chart for fistula remission; **(c)** Bar chart for fistula response; **(d)** Line chart for fistula response].

**Figure 6** presents forest plots comparing each treatment with PBO. For fistula remission, both anti-TNF-α (OR = 2.19, 95% CrI: 1.01–4.54) and MSCs (OR = 5.33, 95% CrI: 2.19–19.03) were significantly superior to PBO (**Figure 6a**). SUCRA rankings suggested that MSCs had the highest probability of being the best intervention for fistula remission: MSCs (0.97) > anti-TNF-α (0.517) > PBO (0.013). Furthermore, the probability of MSCs being the best intervention was 89%, further supporting its advantage in this outcome (**Figures 7a, b**). For fistula response, only MSCs (OR = 4.76, 95% CrI: 1.78–20.37) significantly outperformed PBO, whereas anti-TNF-α (OR = 1.89, 95% CrI: 0.88–3.69) did not reach statistical significance (**Figure 6b**). SUCRA rankings again placed MSCs first:MSCs (0.967) > Anti-TNF-α (0.508) > PBO (0.025) (**Figures 7c, d**).

**Figure 8:**
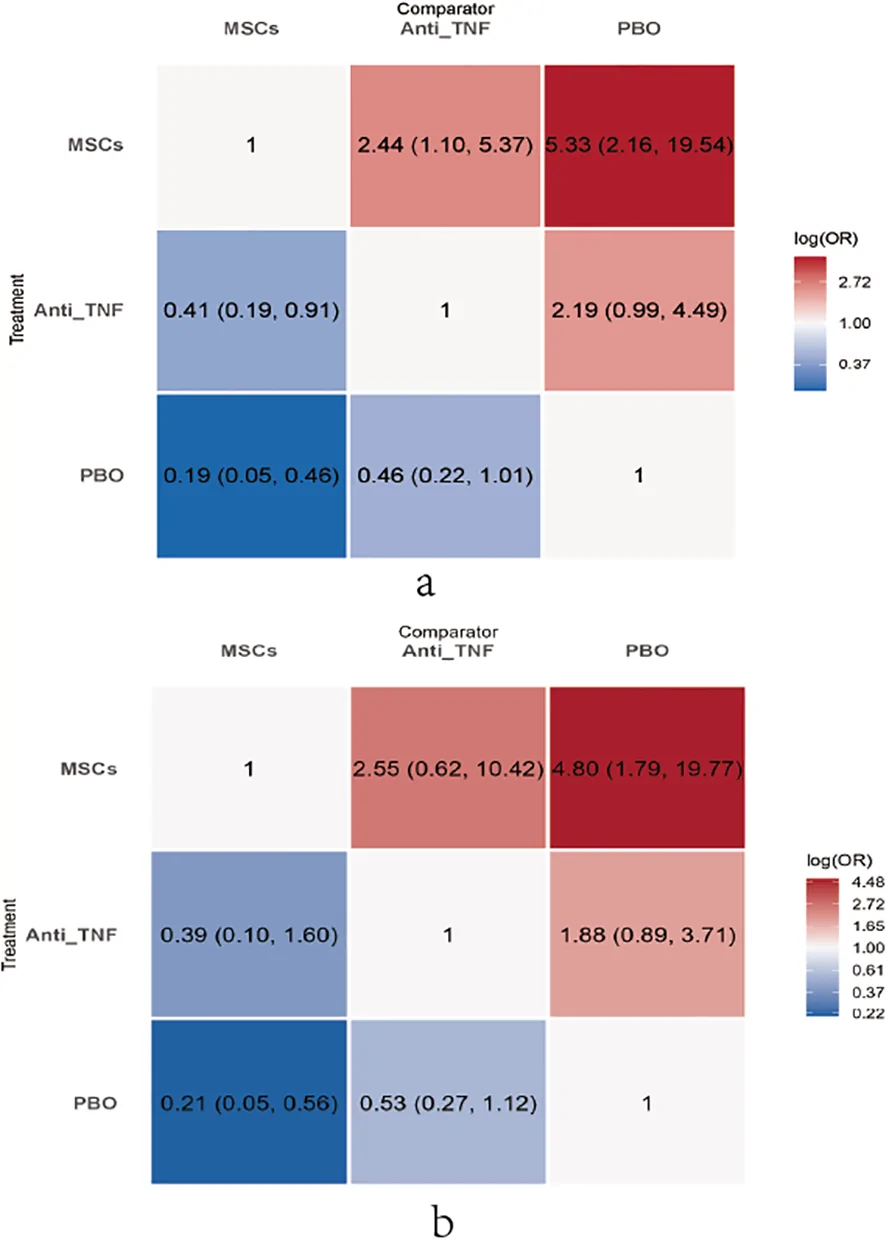
League tables [**(a)** fistula remission; **(b)** fistula response]. (Rows represent reference treatments and columns represent comparator treatments. The matrix is reciprocally symmetric, with values in the lower triangle being the reciprocal of those in the upper triangle. OR > 1 indicates that the row treatment is superior to the column treatment. Treatments are ranked from highest to lowest based on SUCRA values, with the best treatment in the top-left corner and the worst in the bottom-right. Color intensity reflects the magnitude of log(OR): red indicates OR > 1, blue indicates OR < 1, and white indicates OR = 1.).

The league table heatmap (**Figure 8**) further details all pairwise comparisons. For fistula remission induction (**Figure 8a**), both MSCs and anti-TNF-α outperformed PBO, with a larger point estimate for MSCs (OR = 5.33). For fistula response rate (**Figure 8b**), only MSCs significantly surpassed PBO (OR = 4.80). Indirect comparisons between active treatments yielded no statistical significance.

### 3.6 Secondary outcome: clinical remission

**Figure 9:**
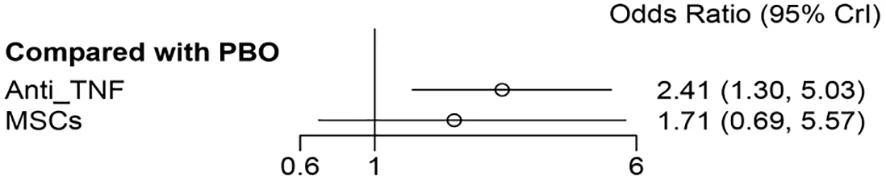
Forest plot for clinical remission.

**Figure 10:**
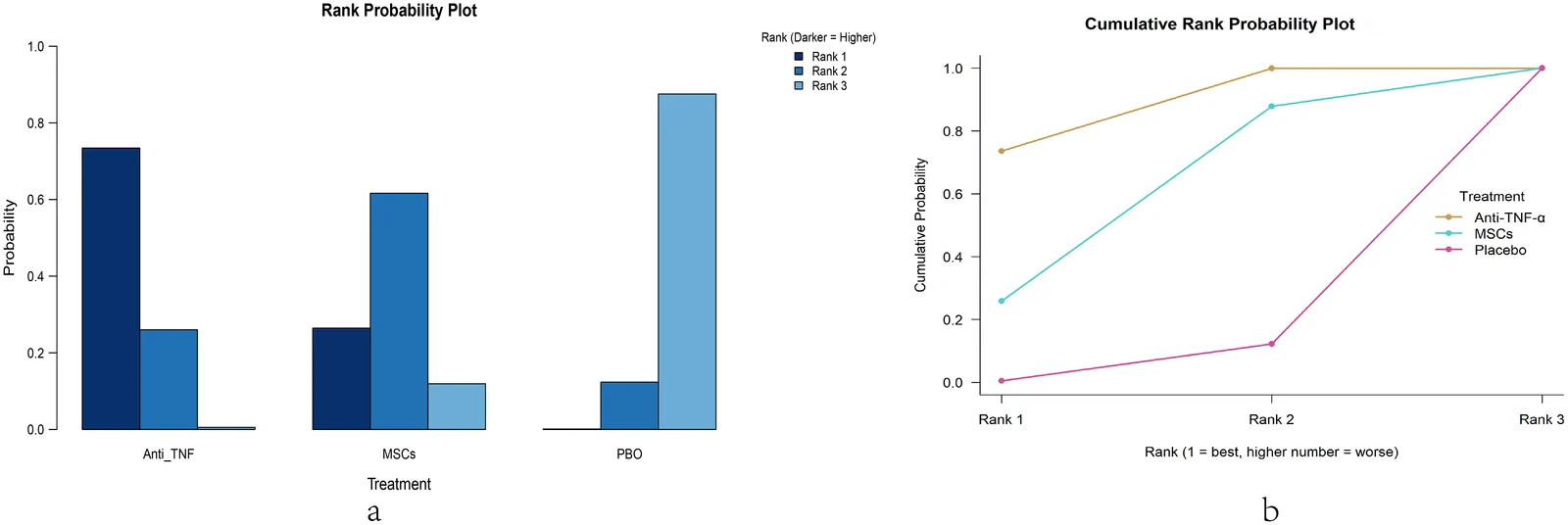
SUCRA ranking plots [**(a)** Bar chart for clinical remission; **(b)** Line chart for clinical remission].

**Figure 11:**
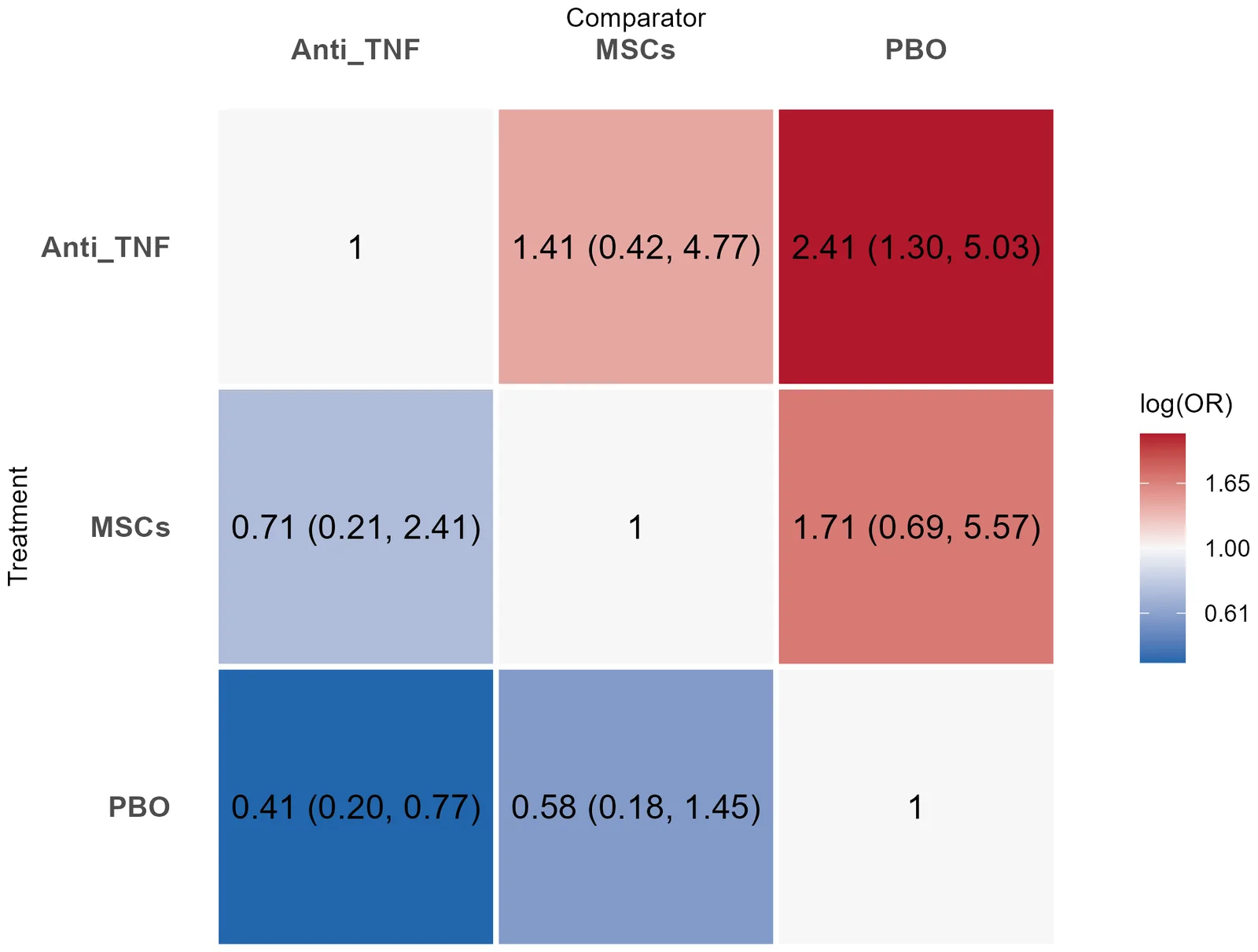
League table for clinical remission.

Both Anti-TNF-α and MSCs outperformed PBO in inducing clinical remission (**Figures 9**, **10**). Anti-TNF-α ranked highest (SUCRA: 0.863), followed by MSCs (0.557) and PBO (0.06). Pairwise comparisons (**Figure 11**) showed that only Anti-TNF-α significantly improved remission rates versus PBO (OR = 2.41, 95% CrI: 1.30–5.03); MSCs did not reach significance (OR = 1.71, 95% CrI: 0.69–5.57). Indirect comparisons indicated comparable efficacy between active treatments (OR = 1.41, 95% CrI: 0.42–4.77).

### 3.7 Safety outcomes: adverse events (AEs and infections)

**Figure 12:**
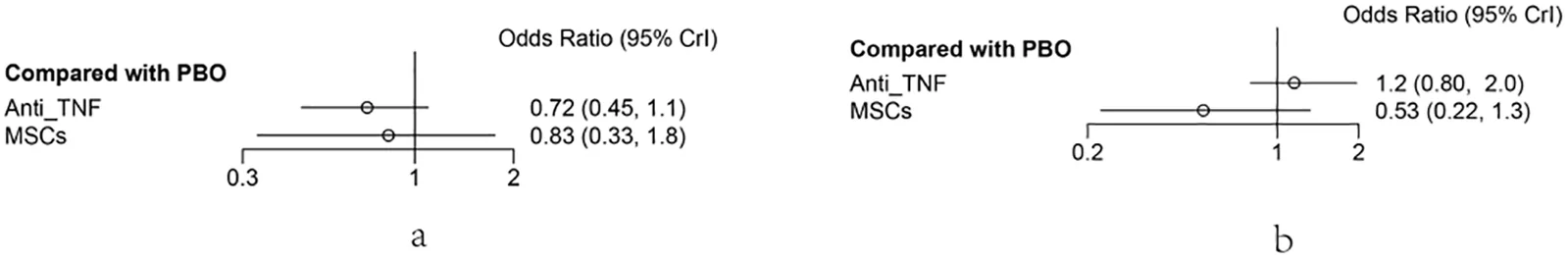
Forest plots [**(a)** AEs; **(b)** Infection rate].

**Figure 13:**
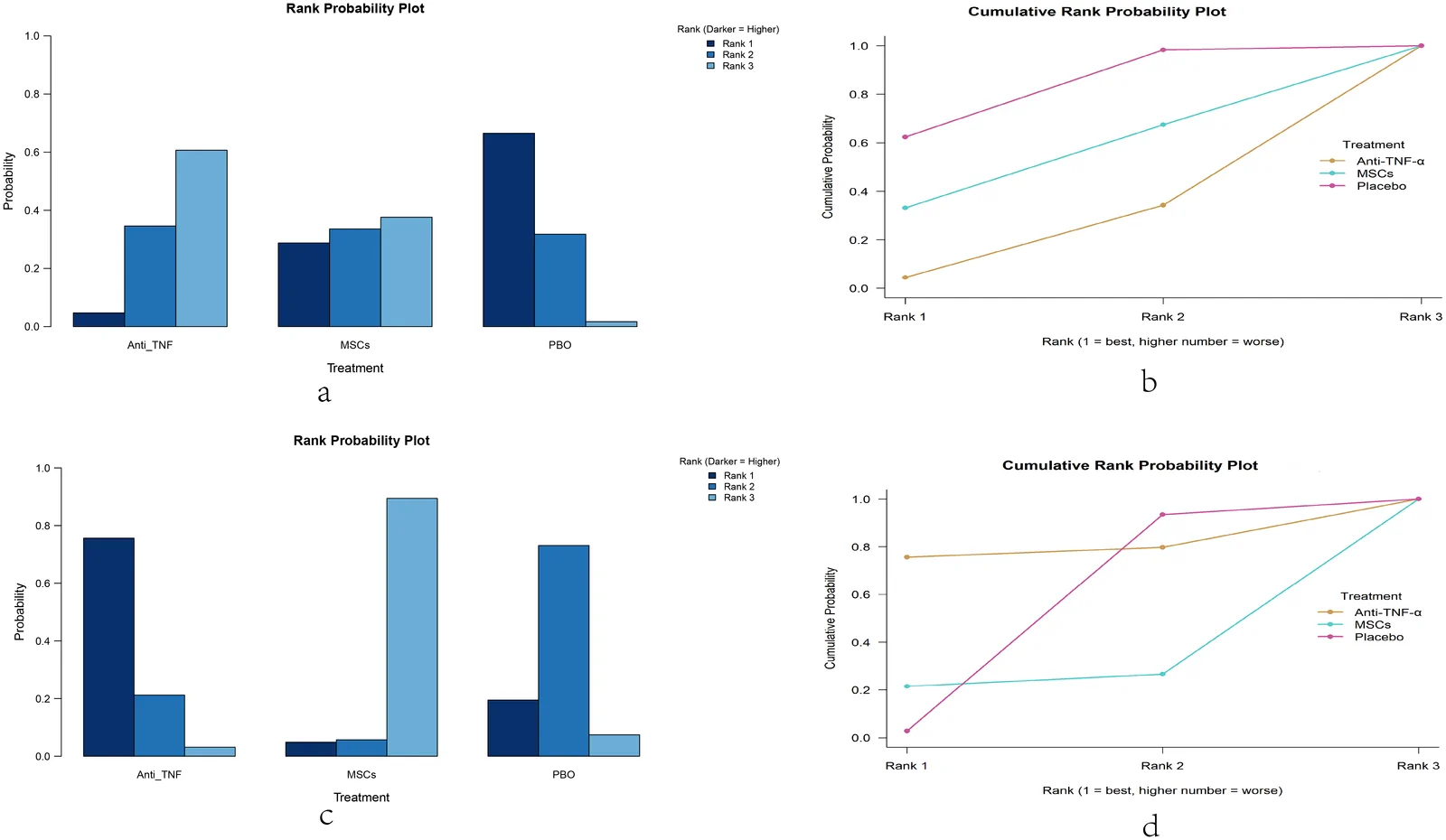
SUCRA ranking plots [**(a** Bar chart for AEs; **(b)** Line chart for AEs; **(c)** Bar chart for infection rate; **(d)** Line chart for infection rate].

**Figure 14:**
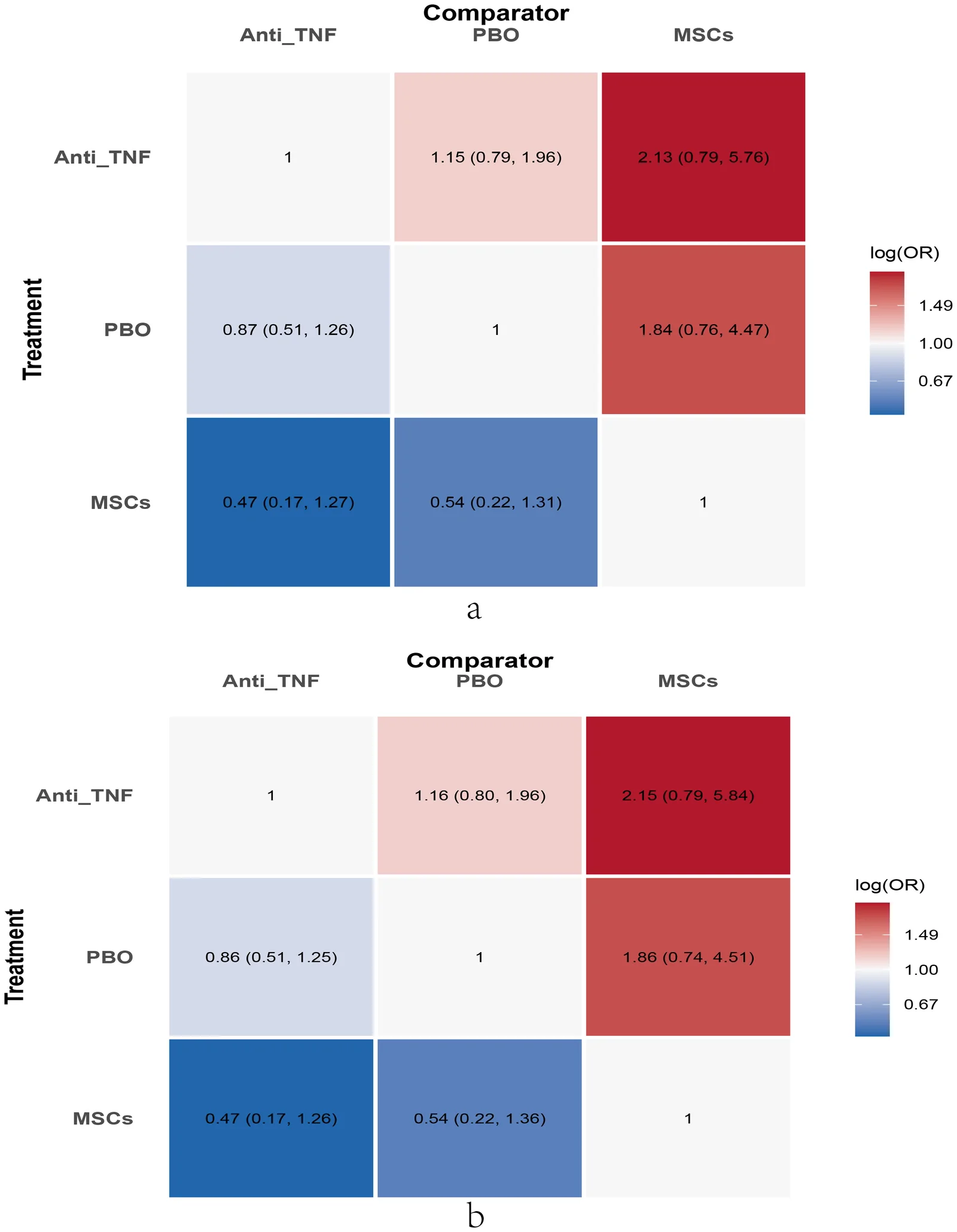
League tables for safety outcomes [**(a)** AEs; **(b)** Infection rate].

Neither Anti-TNF-α nor MSCs significantly differed from PBO in AEs (**Figure 12a**) or infections (**Figure 12b**) (all 95% CrIs included 1), indicating favorable safety profiles. SUCRA rankings for AEs were (**Figure 13a**): PBO (0.82) > MSCs (0.472) > Anti-TNF-α (0.209). Conversely, rankings for infection risk were (**Figure 13b**): Anti-TNF-α (0.865) > PBO (0.562) > MSCs (0.073). League tables (**Figures 14a, b**) further confirmed that all pairwise comparisons yielded odds ratios close to unity, and no statistically significant differences were observed among any of the treatment groups.

### 3.8 Model convergence

Convergence of the Bayesian random-effects network meta-analysis was assessed via trace plots, posterior density plots, and potential scale reduction factor (PSRF). Visual inspection showed MCMC chains stabilized after 200, 000 iterations without divergence or cycling. All PSRF values ranged from 1.00 to 1.02 (<1.05), confirming convergence. Trace plots demonstrated adequate mixing with no autocorrelation, while symmetric, unimodal density plots indicated stable posterior distributions (**Supplementary File 3**, **Supplementary Figures 1**-**5**).

### 3.9 Certainty of evidence

We did not perform a formal GRADE assessment for each outcome. However, the certainty of the evidence was evaluated through the following complementary approaches: (1) the methodological quality of included studies was assessed using the Cochrane Risk of Bias 2.0 tool (**Figures 2**, **3**); (2) heterogeneity was quantified using the I² statistic and τ estimates; (3) precision of effect estimates was assessed through the width of the 95% credible intervals; and (4) publication bias was evaluated using funnel plots and Egger’s test (**Figure 4**). Based on these assessments, the evidence for fistula remission and response (MSCs vs placebo) is of moderate certainty, while the evidence for anti-TNF-α vs placebo for fistula response is of lower certainty due to imprecision. A detailed summary of the evidence assessment is provided in **Supplementary File 2**, **Supplementary Table 3**.

## 4 Discussion

PFCD heavily burden patients, accounting for approximately 54% of all CD-related fistulas. Risk factors for fistulizing CD include colonic involvement, male sex, age, prolonged disease duration, and extraintestinal manifestations (40). PFCD pathogenesis is complex and incompletely understood. Epithelial-mesenchymal transition (EMT) drives fibrosis and fistula formation (41), alongside inflammation (42, 43), elevated matrix metalloproteinase (MMP) activity causing extracellular matrix degradation (44), and NOD2/autophagy gene defects (45).Current evidence suggests that in genetically susceptible individuals with impaired mucosal immunity, dysregulated immune responses trigger cytokines (e.g., TNF-α, IL-13, TGF-β), promoting fistula initiation and chronicity (46–48).

For most PFCD patients, the primary treatment goals are symptom relief, fistula closure, and improved quality of life (49). PFCD management is highly challenging. Surgical or medical monotherapies often lead to recurrence, while antibiotics and immunomodulators have limited standalone efficacy. In contrast, anti-TNF-α agents have the most robust evidence as first-line therapy (40). Recently, MSCs have been shown to promote fistula healing through multiple mechanisms (50–54). A recent Phase I/II trial demonstrated significant efficacy of MSC-derived extracellular vesicles (EVs) or exosomes for refractory perianal fistulas (55). However, the lack of head-to-head RCTs comparing anti-TNF-α and MSCs precludes definitive direct efficacy comparisons.

This study systematically compared the efficacy and safety of anti-TNF-α and MSCs for PFCD by Bayesian network meta-analysis. We observed a trend favoring MSCs over in promoting fistula remission and fistula response: Our analysis found that MSCs were significantly superior to placebo in promoting fistula remission (OR = 5.33, 95% CrI: 2.19–19.03) and fistula response (OR = 4.76, 95% CrI: 1.78–20.37). MSCs ranked first in SUCRA for both remission (0.97) and response (0.967), with an 89% probability of being the optimal intervention. Although anti-TNF-α was also significantly superior to placebo (remission OR = 2.19, 95% CrI: 1.01–4.54), its effect size was notably smaller than that of MSCs. This aligns with previous literature. The Phase III ADMIRE-CD trial by Panés et al. demonstrated that local injection of allogeneic adipose-derived MSCs (darvadstrocel) yielded a significantly higher combined remission rate for PFCD than PBO (50% vs. 34%) (24). Furthermore, 52-week long-term follow-up confirmed that clinical remission induced by MSCs can be sustained for up to 104 weeks (56). A recent systematic review and meta-analysis encompassing 25 studies and 596 PFCD patients demonstrated that MSC therapy achieved a combined remission rate of 57.9% (95% CI: 51.3–64.2) at 6 months, with no significant difference observed between adipose-derived and bone marrow-derived MSCs (RR = 0.74, 95% CI: 0.31–1.77) (21). These results are in agreement with prior studies.

Compared with MSCs, anti-TNF-α demonstrated superior efficacy in achieving overall clinical remission (CDAI <150) (OR = 2.41, 95% CrI 1.30–5.03). This reflects their distinct therapeutic profiles: anti-TNF-α rapidly control intestinal inflammation via systemic suppression of inflammatory cytokines (e.g., TNF-α, IL-6), thereby alleviating systemic symptoms (15); Conversely, MSCs primarily focus on local tissue repair and immunomodulation at the fistula site, exhibiting a relatively weaker effect on systemic inflammation (57). Thus, Our findings offer practical guidance for PFCD management. MSCs may be particularly beneficial for patients with complex or refractory fistulas, or those intolerant to anti-TNF-α, by providing local tissue repair where systemic agents have limited penetration. Anti-TNF-α remains the first-line choice for patients with active luminal disease, given its superior ability to induce clinical remission. A sequential approach—initiating anti-TNF-α for systemic control followed by adjunctive MSC injection for durable fistula closure—may represent an optimized strategy. In the absence of direct comparative evidence, treatment decisions should be individualized based on disease phenotype, prior therapy, and patient preference.

Our indirect comparison showed no statistically significant differences between MSCs and anti-TNFα inhibitors in fistula remission (OR = 2.44) and response (OR = 2.55). This suggests comparable efficacy, although MSCs may offer advantages in specific subgroups, such as complex fistulas or anti-TNFα failures. However, all comparisons between active treatments are indirect and rely on the assumption of transitivity. The SUCRA rankings cannot constitute proof of superiority in the absence of direct randomized evidence, while useful for hypothesis generation. This conclusion requires further validation through head-to-head randomized controlled trials.

The therapeutic mechanisms of MSCs are likely attributed to their unique immunomodulatory and tissue-repair properties. Through paracrine signaling, MSCs secrete anti-inflammatory factors to suppress overactive Th1/Th17 cells and inhibit the release of pro-inflammatory cytokines (e.g., TNF-α, IFN-γ). Concurrently, they induce regulatory T cell differentiation and promote angiogenesis alongside fibrotic repair in damaged tissues (58, 59). Additionally, MSCs alleviate local inflammation by inhibiting neutrophil and macrophage infiltration, thereby creating a favorable microenvironment for fistula healing (60, 61). MSC-derived angiogenic and epidermal growth factors directly stimulate fibroblast and epithelial cell proliferation, accelerating mucosal repair and granulation tissue formation at fistula defects. Additionally, MSCs promote neovascularization to alleviate local ischemia and hypoxia, thereby providing essential nutritional support for tissue regeneration (62, 63). Recent studies have confirmed that MSCs exert their therapeutic effects primarily through paracrine pathways rather than direct differentiation into tissue cells. As key paracrine mediators, exosomes have become a focal point of mechanistic research (55).

However, variations in MSC sources (adipose tissue vs. bone marrow), dosages (1×10^7^ to 12×10^7^ cells), and administration regimens, follow-up time points likely account for the high heterogeneity observed in this study (I² = 72.8%). Therefore, we should be cautious when extrapolating these findings to routine clinical practice. The pooled effect estimate reflects the average effect of highly heterogeneous MSC products and treatment protocols, rather than a stable and reproducible efficacy. This is particularly relevant given that even the same MSC product yielded inconsistent results in the ADMIRE-CD (24) and ADMIRE-CD II (64) trials, underscoring the impact of patient selection and perioperative management on treatment outcomes. Although the favorable direction of the effect and the SUCRA ranking of MSCs were consistent across analyses, the substantial heterogeneity (I²=72.8%) warrants caution in interpreting the pooled effect estimate as a reliable measure of efficacy for all MSC preparations. Future studies should standardize MSC preparation and administration protocols to minimize the impact of heterogeneity on outcomes.

TNF-α sustains a pro-inflammatory milieu, whereas anti-TNF-α bind to TNF-α to neutralize its biological effects. They suppress the immune cascade by inhibiting inflammatory cytokines and promoting anti-inflammatory responses (65). Previous studies have also established the definitive efficacy of infliximab in inducing and maintaining fistula closure in CD, with complete closure rates reaching 46%–55% (8). A 2023 network meta-analysis showed infliximab (5 mg/kg) achieved optimal efficacy at 16–24 weeks for fistulizing CD (RR = 2.30, 95% CI 1.40–3.77) (66).

However, anti-TNF-α therapy is limited by high non-response and relapse rates. About 50%–60% of patients fail to achieve complete fistula closure post-induction, leading to poor long-term maintenance (8, 67). The PISA-II trial (median 5.7-year follow-up) showed only 18% radiological healing with anti-TNF-α monotherapy, and a 36% relapse rate among clinically healed patients (17). Although effective for systemic inflammation, anti-TNF-α face local penetration barriers in complex perianal fistulas. Chronic fibrosis and sphincter edema hinder the diffusion of large monoclonal antibodies, leaving the core inflammatory microenvironment unaddressed (68). Thus, MSCs may offer a viable alternative or adjunctive therapy for anti-TNF-α failures or intolerant patients.

Notably, the star-shaped network structure of this study (where all interventions were compared solely against PBO) precluded consistency testing using the node-splitting method. All indirect comparisons rely on the assumption of transitivity, which posits that studies in direct comparisons are similar in terms of population characteristics, outcome definitions, and effect modifiers (69).

The safety of MSCs and anti-TNF-α for PFCD remains debated. However, our study found no significant differences from PBO in adverse events or infections for either therapy, indicating a favorable safety profile consistent with previous meta-analyses (70–72). A meta-analysis showed no significant difference in adverse events between the MSCs and control groups (OR = 1.06, 95% CI 0.68–1.59), with no events attributed to MSCs (73), Another meta-analysis found that anti-TNF-α therapy significantly reduced the risk of serious adverse events (SAEs) compared to controls (OR = 0.80, 95% CI 0.67–0.96) (74). Notably, anti-TNF-α seemed to rank better than PBO in infection control, an unexpected finding likely attributable to varying definitions of infectious events, differing follow-up durations, or disparate baseline infection risks across the original studies. In the ACCENT 2 trial, cumulative infliximab exposure was not associated with fistula-related abscesses (8), Furthermore, while anti-TNF-α therapy shows greater efficacy in studies where fistula response is the primary endpoint, its safety profile requires validation in larger cohorts (16). Common adverse events (e.g., anal pain, fever, abdominal pain, and perianal abscesses) may be related to the injection procedure rather than the drug itself. Future long-term follow-up of larger patient populations is essential to comprehensively evaluate the safety of MSCs for PFCD.

Beyond anti-TNF-α and MSCs, several emerging and adjunctive therapies are under investigation for refractory PFCD. Exosomes have been shown to achieve complete closure of refractory perianal fistulas in 60% of patients and 69.7% of fistulas at six months (55). Among these, hyperbaric oxygen therapy (HBOT) has shown remarkable efficacy in selected patients with refractory PFCD. A study by Lansdorp et al. demonstrated significant improvements in patients treated with HBOT after failing standard interventions (75). Literatures reported that combining hyperbaric oxygen therapy with medications has improved patient response rates (76, 77). Other emerging approaches include novel small molecules, such as Janus kinase inhibitors, IL-23 Inhibitors, Anti-α4β7 Integrin Agents, and anti-TNF-α combined with surgical seton placement or local stem cell therapy, also represent promising avenues. These therapies offer potential alternatives for patients who fail to respond to current first-line options.

In addition to the intrinsic constraints of individual studies, our analysis has some limitations: First, the number of included studies is limited. We pooled 15 RCTs (2, 475 patients), but only seven evaluated MSCs (412 patients). Small sample sizes in some studies limited the precision of effect estimates. Moreover, the small number of studies precluded subgroup analyses and meta-regression to explore heterogeneity; Second, substantial clinical heterogeneity exists regarding fistula types, MSC sources, and dosages across studies, potentially affecting result heterogeneity and generalizability. Future studies should adopt standardized inclusion criteria and intervention protocols. Finally, limitations also include a lack of radiological assessments and incomplete safety data. The reliance of primary outcomes on clinical evaluation, without standardized imaging assessments (e.g., MRI), may overestimate the durability of clinical healing. Furthermore, the variability in follow-up durations across studies makes it difficult to assess long-term rare adverse events.

Despite these limitations, this study adds to our understanding of one of the most clinically important concerns related to PFCD medication. It represents the first application of Bayesian network meta-analysis to quantitatively compare the relative efficacy of anti-TNF-α and MSCs within a unified framework, bridging the gap left by traditional pairwise meta-analyses that cannot directly compare different intervention categories. It also systematically elucidates, the differential efficacy profiles of these two therapeutic approaches, providing a stratified basis for individualized treatment strategies. Besides, our study comprehensively integrates the latest evidence on MSC therapy for PFCD, while identifying key evidence gaps, thus pointing directions for future research. Furthermore, to better guide the relative positioning of recently available drugs in clinical practice, meaningful head-to-head trials in PFCD patients are required.

## 5 Conclusion

Our Bayesian network meta-analysis shows that both MSCs and anti-TNF-α are effective in treating PFCD, with complementary efficacy profiles. While anti-TNF-α was superior to placebo in inducing clinical remission, MSCs demonstrated a numerically higher but statistically non-significant point estimate for fistula healing in indirect comparisons against anti-TNF-α. Both have favorable safety profiles, making MSCs a viable alternative for patients failing or intolerant to anti-TNF-α therapy. Future head-to-head RCTs and long-term follow-ups are warranted to validate these findings and explore combination therapies.

## Data Availability

The original contributions presented in the study are included in the article/**Supplementary Material**. Further inquiries can be directed to the corresponding author.
