# Supplementary material for: Efficacy and safety of tumor necrosis factor-α inhibitors and mesenchymal stem cells in the treatment of fistulizing Crohn’s disease: a systematic review and network meta-analysis
Source: Front Immunol. 2026 Sep 11;17:1929235. doi: 10.3389/fimmu.2026.1929235 (PMC13612554; doi:10.3389/fimmu.2026.1929235)
Supplement: Supplementary file 1 [file DataSheet2.pdf]

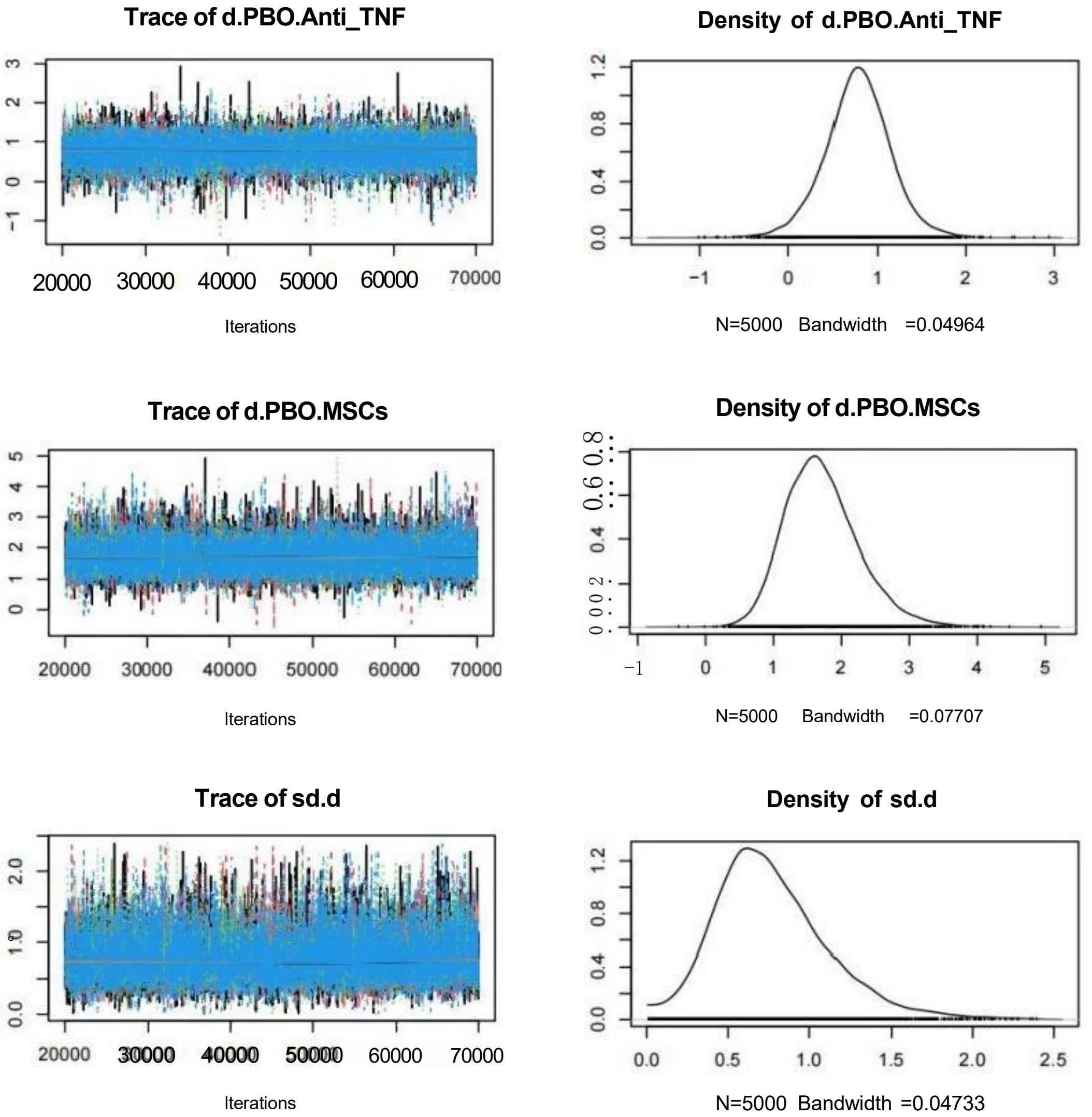

Fig S1. Fistula Remission Rate Trace and Density Plot

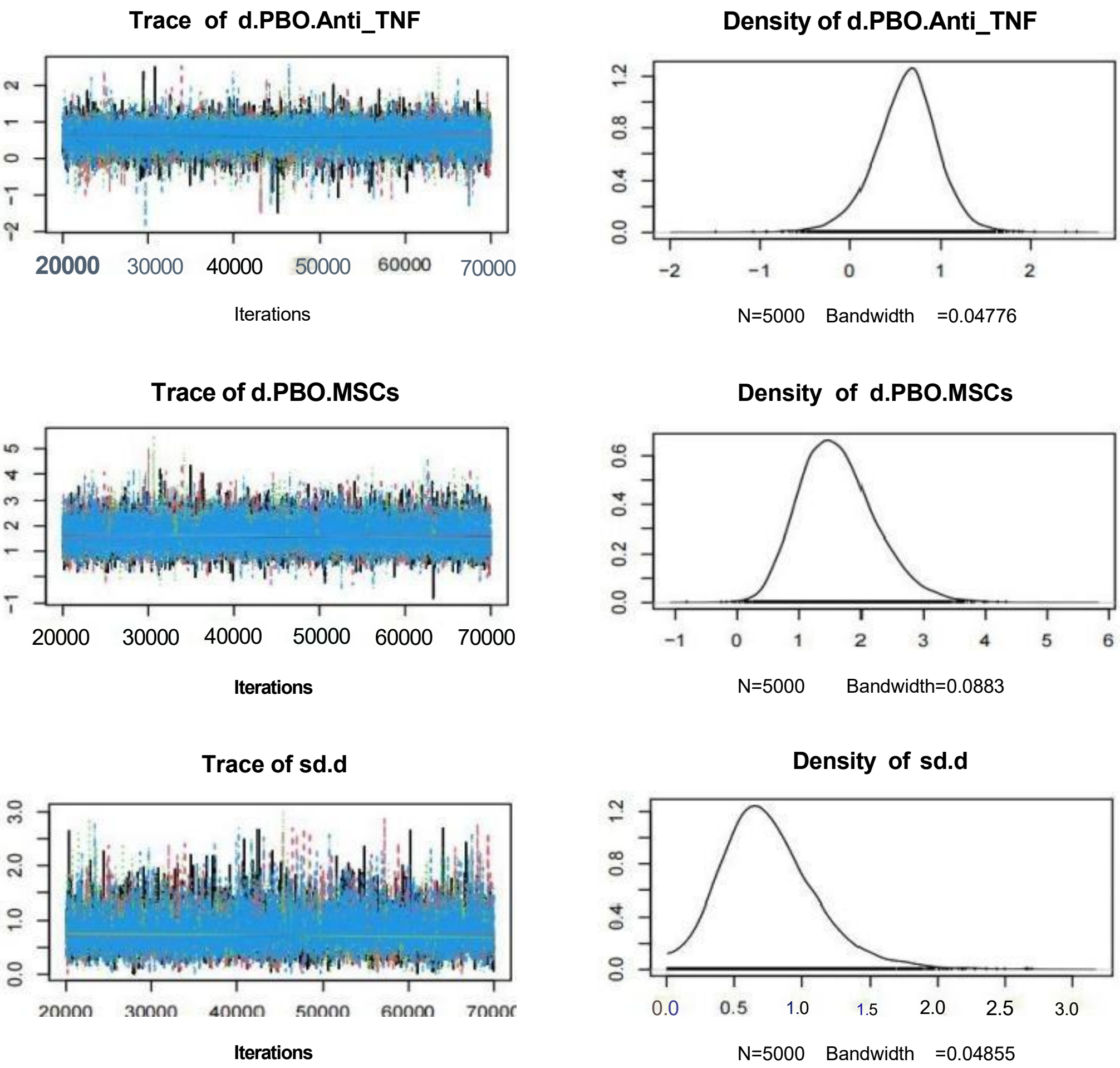

Fig S2. Fistula Response Rate Trace and Density Plot

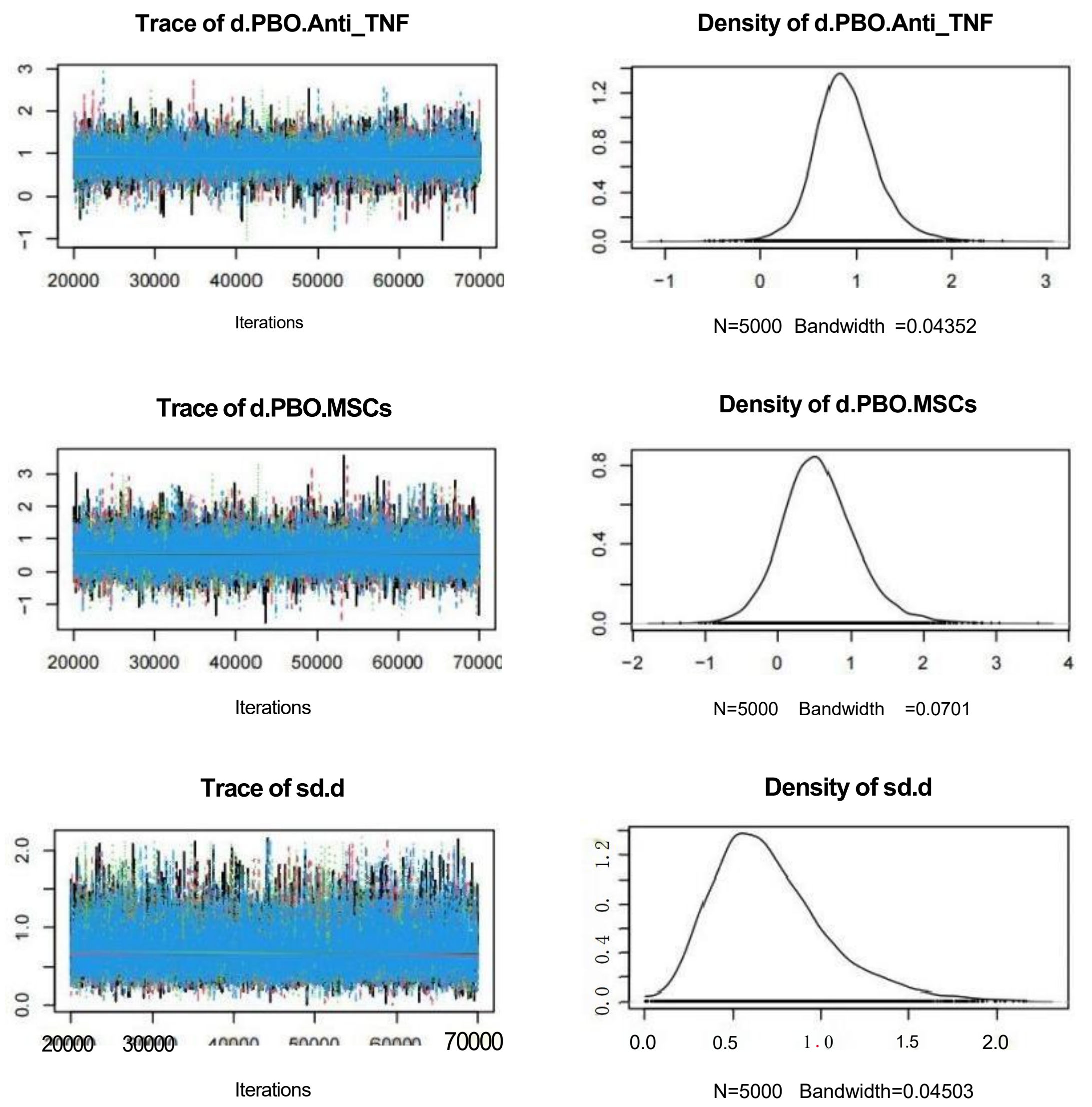

Fig S3. Clinical Remission Rate Trace and Density Plot

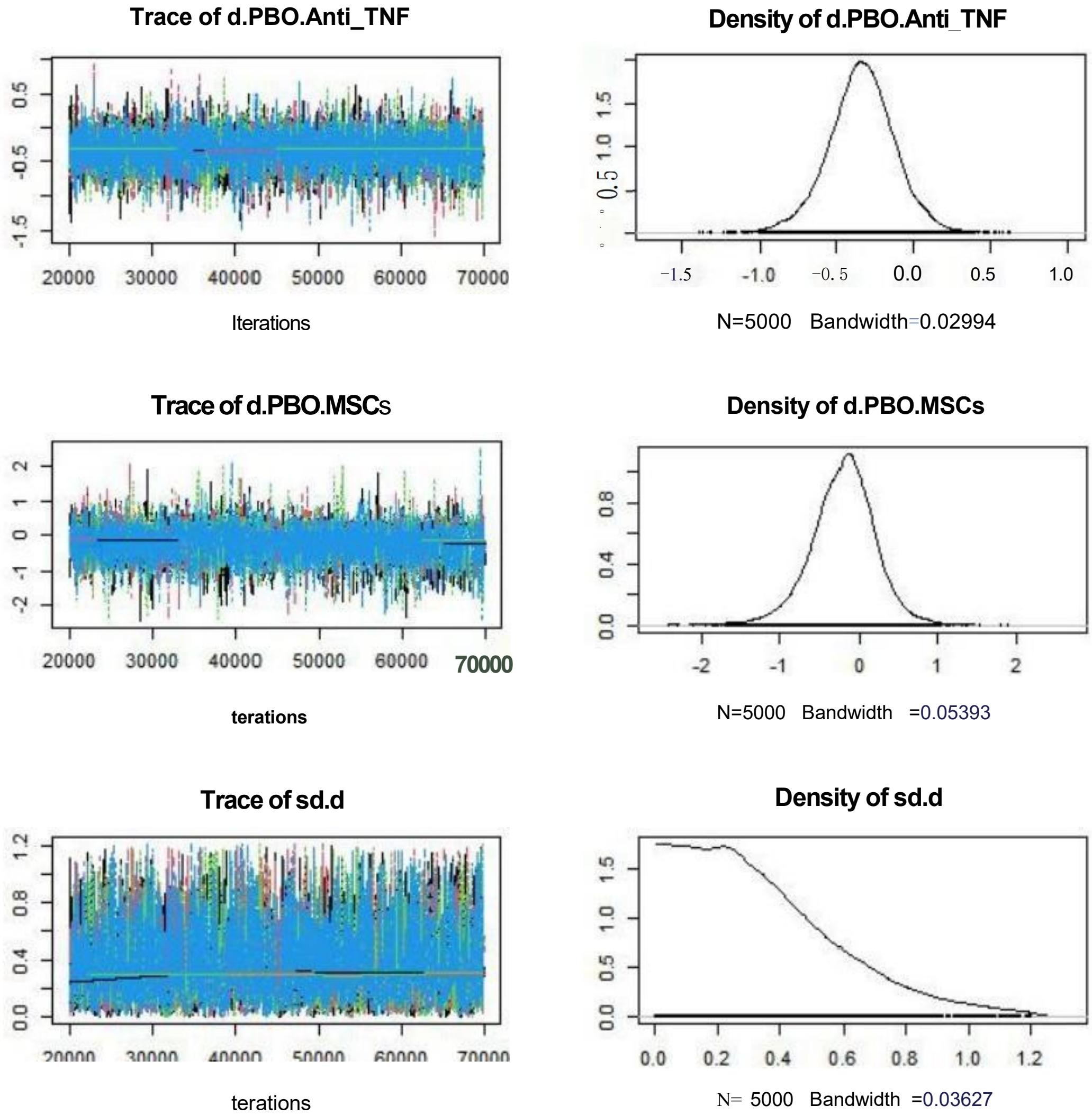

Fig S4. Adverse Events Rate Trace and Density Plot

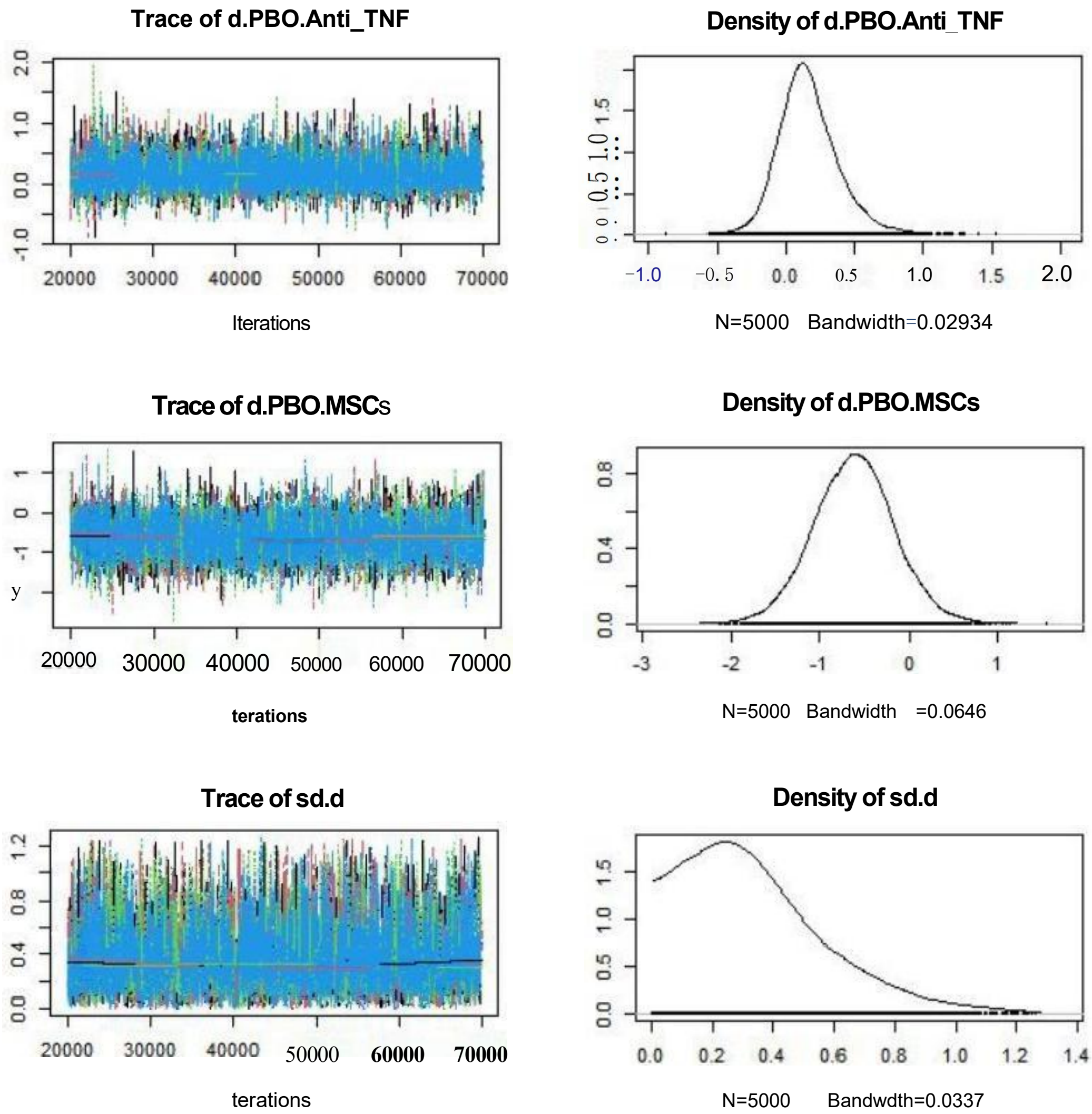

Fig S5. Infection Rate Trace and Density Plot
