# Supplementary material for: Efficacy and safety of tumor necrosis factor-α inhibitors and mesenchymal stem cells in the treatment of fistulizing Crohn’s disease: a systematic review and network meta-analysis
Source: Front Immunol. 2026 Sep 11;17:1929235. doi: 10.3389/fimmu.2026.1929235 (PMC13612554; doi:10.3389/fimmu.2026.1929235)
Supplement: Supplementary file 2 [file DataSheet1.docx]

Supplementary File 1

search strategy

\# Web of Science search strategy (v0.1)

\# search :

1: TS=(infliximab OR "Monoclonal Antibody cA2" OR ("cA2" NEAR/3 ("Monoclonal" OR "MAb")) OR remicade OR	inflectra OR renflexis OR "infliximab-dyyb" OR "infliximab dyyb" OR	"infliximab-abda" OR	"infliximab abda")	Results: 29320

2: TS=( (crohn* AND (disease* OR enteritis OR colitis OR ileitis)) OR "regional enteritis" OR	("granulomatous enteritis" OR "enteritis, granulomatous") OR	("regional ileitis" OR "ileitis, regional") OR	"terminal ileitis" OR

("granulomatous colitis" OR "colitis, granulomatous") OR	ileocolitis OR	"inflammatory bowel disease 1" OR

ibd1)	Results : 80670

3: TS=(	"anal fistul*" OR	"anorectal fistul*" OR	"perianal fistul*" OR

("ano*" NEAR/3 fistul*) OR	"fistula-in-ano")	Results: 4898

4: #2 AND #3	Results: 1925

5: TS=(	("Tumor Necrosis Factor alpha" OR "Tumor Necrosis Factor-alpha") OR	("TNF-alpha" OR "TNFalpha") OR	"Cachectin" OR

("Cachectin Tumor Necrosis Factor" OR "Cachectin-Tumor Necrosis Factor") OR	("TNF Superfamily, Member 2" OR "Tumor Necrosis Factor Ligand Superfamily Member 2"))	Results: 249088

6: TS=(	adalimumab OR	humira OR	"D2E7 Antibody" OR	("D2E7" NEAR/3 ("Antibody" OR "mAb" OR "Monoclonal")) OR	amjevita OR

cyltezo OR	"adalimumab-atto" OR	"adalimumab atto" OR	"adalimumab-adbm" OR	"adalimumab adbm" OR	"adalimumab-*")	Results: 16945

7: TS=(	golimumab OR	simponi OR	"CNTO-148" OR	"CNTO 148")	Results: 2310

8: TS=( "Certolizumab Pegol" OR	cimzia OR	"CDP-870" OR	"CDP870" OR	"CDP 870")	Results: 2429

9: TS=(	etanercept OR	enbrel OR	erelzi OR	"etanercept-szzs" OR

"TNF Receptor Type II-IgG Fusion Protein" OR	"TNF Receptor Type II IgG Fusion Protein" OR	"TNFR-Fc Fusion Protein" OR

("TNFR" NEAR/3 "Fc" NEAR/3 "Fusion Protein") OR	("TNTR-Fc" OR "TNR-001" OR "TNR 001" OR "TNR001") OR	"TNT Receptor fusion Protein" OR	"Recombinant Human Dimeric TNF Receptor Type II-IgG Fusion Protein")	Results: 13534

10: TS=(	etanercept OR	enbrel OR	erelzi OR	"etanercept-szzs" OR	"TNF Receptor Type II-IgG Fusion Protein" OR	"TNF Receptor Type II IgG Fusion Protein" OR	"TNFR-Fc Fusion Protein" OR

("TNFR" NEAR/3 "Fc" NEAR/3 "Fusion Protein") OR	("TNTR-Fc" OR "TNR-001" OR "TNR 001" OR "TNR001") OR	"TNT Receptor fusion Protein" OR	"Recombinant Human Dimeric TNF Receptor Type II-IgG Fusion Protein")	Results: 13534

11: TS=(("mesenchymal stem cell*" OR "stem cell*, mesenchymal") OR ("mesenchymal stromal cell*" OR "stromal cell*, mesenchymal") OR	("mesenchymal progenitor cell*" OR "progenitor cell*, mesenchymal") OR	("multipotent mesenchymal stromal cell*" OR "multipotent stromal cell*" OR "mesenchymal stromal cells, multipotent") OR	("bone marrow stromal cell*" OR "bone marrow mesenchymal stem cell*" OR "multipotent bone marrow stromal cell*" OR "bone marrow stromal stem cell*") OR	("adipose-derived mesenchymal stem cell*" OR "adipose derived mesenchymal stem cell*" OR "adipose-derived mesenchymal stromal cell*" OR "adipose derived mesenchymal stromal cell*" OR "mesenchymal stem cells, adipose-derived" OR "mesenchymal stem cells, adipose derived") OR	("adipose tissue-derived mesenchymal stem cell*" OR "adipose tissue derived mesenchymal stem cell*" OR "adipose tissue-derived mesenchymal stromal cell*" OR "adipose tissue derived mesenchymal stromal cell*") OR ("wharton* jelly cell*" OR "wharton's jelly cell*" OR "whartons jelly cell*"))	Results: 129916

12: #5 OR #6 OR #1 OR #7 OR #8 OR #9	Results: 288471

13: #11 OR #12	Results: 413910

14: #4 AND #13	Results: 748
