# Supplementary material for: Efficacy and safety of tumor necrosis factor-α inhibitors and mesenchymal stem cells in the treatment of fistulizing Crohn’s disease: a systematic review and network meta-analysis
Source: Front Immunol. 2026 Sep 11;17:1929235. doi: 10.3389/fimmu.2026.1929235 (PMC13612554; doi:10.3389/fimmu.2026.1929235)
Supplement: Supplementary file 3 [file DataSheet3.docx]

Table S1 Baseline Characteristics of Included Studies

| Study | Sample(n) | | Age (years, mean ± SD) | | Proportion of males(%) | | CDAI | |
| --- | --- | --- | --- | --- | --- | --- | --- | --- |
|  | Intervention | Comparator | Intervention | Comparator | Intervention | Comparator | Intervention | Comparator |
| Garcia-Olmo （2009） | 15 | 6 | 42.64 ± 10.93 | 43.99 ± 8.97 | 42% | 56% | NA | NA |
| Molendijk（2015） | 107 | 105 | 38.2 ± 8.5 | 37.3 ± 8.8 | Group 1: 80.0% Group 2: 80.0% Group 3: 20.0% | 50.0% | Group 1 : 80.2 ± 12.1 Group 2 : 203.3 ± 51.2 Group 3 : 57.3 ± 14.1 | 75.8±28.2 |
| Panés（2016） | 16 | 6 | 39.0 ± 13.1 | 37.6 ± 13.1 | 56% | 53% | 88.7 ± 48.8 | 94.2 ± 58.7 |
| Lightner（2023Ⅰ) | 18 | 5 | median age: 41.2 | | NA | NA | 11.0±5.4# | 10.0±3.0# |
| Lightner（2023Ⅱ） | 15 | 4 | 34.0±12.9 | 33.6±16.3 | 38.90% | 80% | NA | NA |
| Lightner（2024） | 15 | 6 | 40.8±12.1 | 49.2±17.0 | 0% | 0% | NA | NA |
| Barnhoorn（2019） | 63 | 31 | **Median (range): 42.0 (**26.0–57.0) | NA | Group 1: 75%  Group 2: 100%  Group 3: 20% | NA | 101.5 ± SEM | NA |

| Present（1999） | 263 | 132 | 35.9 ± 11.5 | 36.5 ± 10.4 | 42.90% | 55% | **Median (INQ):** 168(112–258） | **Median (INQ):** 162( 126–265） |
| --- | --- | --- | --- | --- | --- | --- | --- | --- |
| Sandborn（2004） | 91 | 98 | 37.9 ± 12.4 | 37.7 ± 12.5 | 39.90% | 32.60% | 297 ± 63 | 301 ± 61 |
| Sands（2004） | 225 | 74 | **Median (range):** 36 .0(29.0–46.0) | **Median (range):** 37 .0(28.0–47.0) | 55% | 48% | NA | NA |
| Hanauer（2006） | 159 | 166 | 38.7 ± 12.0 | 37.0 ± 13.0 | 44.40% | 50% | 298.3 ± 56.7 | 296 ± 60 |
| Sandborn（2007Ⅰ） | 331 | 328 | 39.0 ± 12.0 | 37.0 ± 12.0 | 31% | 39% | 313 ± 58 | 313 ± 66 |
| Sandborn（2007Ⅱ） | 70 | 47 | 37.0 ± 12.0 | 38 .0± 12.0 | 47% | 40% | 300 ± 64 | 297 ± 62 |
| Colombel（2009） | 28 | 30 | 35.9 ± 12.2 | 36.5 ± 10.1 | 48.6% | 31.9% | 318.4 ± 55.8 | 308.0 ± 59.3 |
| Schreiber（2011） | 15 | 6 | 37.3 ± 9.4 | 37.6 ± 10.1 | 50% | 67% | 299.8 ± 58.3 | 317.1 ± 69.4 |

Table S2：Heterogeneity Results of the Meta-analysis

| Outcomes | Direct comparisons | Study Count | I^2^ |
| --- | --- | --- | --- |
| Fistula remission | Anti-TNFα vs PBO | 8 | 32.5% |
|  | MSCs vs PBO | 7 | 72.8% |
| Fistula response | Anti-TNFα vs PBO | 8 | 48.6% |
|  | MSCs vs PBO | 7 | 69.3% |
| Clinical remission | Anti-TNFα vs PBO | 7 | 71.8% |
|  | MSCs vs PBO | 5 | 59.9% |
| AEs | Anti-TNFα vs PBO | 8 | 7.6% |
|  | MSCs vs PBO | 5 | 0.0% |
| Infection rate | Anti-TNFα vs PBO | 8 | 33.2% |
|  | MSCs vs PBO | 6 | 0.0% |

Table S3：Heterogeneity Results of the Meta-analysis

| Outcome | Comparison | Certainty | Reason for downgrading |
| --- | --- | --- | --- |
| Fistula remission | MSCs vs PBO | Moderate | Inconsistency (I² = 72.8%) |
| Fistula remission | Anti-TNF-α vs PBO | Moderate | Imprecision (wide CI) |
| Fistula response | MSCs vs PBO | Moderate | Inconsistency |
| Fistula response | Anti-TNF-α vs PBO | Low | Imprecision + indirectness |
| Clinical remission | Anti-TNF-α vs PBO | Moderate | Imprecision |
| Clinical remission | MSCs vs PBO | Low | Imprecision + indirectness |
| Adverse events | MSCs vs PBO | High | None |
| Adverse events | Anti-TNF-α vs PBO | High | None |
| Infections | MSCs vs PBO | Moderate | Imprecision (low events) |
